# Integrative In Vivo and Proteomic Analysis of a *Bovistella utriformis* Polysaccharide Formulation Reveals Mechanisms of Enhanced Skin Wound Healing

**DOI:** 10.3390/molecules31081233

**Published:** 2026-04-08

**Authors:** Aya Maaloul, Juan Decara, Piedad Valverde-Guillén, Casimiro Cárdenas-García, Cristian Riquelme, Claudia Pérez Manríquez, Antonio Jesús López-Gambero, María Albendea Santana, Manuel Marí-Beffa, Marisel Araya-Rojas, Victor Fajardo, Roberto Teófilo Abdala-Díaz

**Affiliations:** 1 Department of Ecology and Geology, Faculty of Science, University of Málaga, 29071 Malaga, Spain; maalouleya6@gmail.com (A.M.); malbendea02@gmail.com (M.A.S.); 2 Grice Hutchinson Experimental Centre, Institute of Blue Biotechnology and Development (IBYDA), University of Málaga, Lomas de San Julián, 29004 Malaga, Spain; piedad.vg.10@uma.es (P.V.-G.); beffa@uma.es (M.M.-B.); 3 Instituto de Investigación Biomédica de Málaga y Plataforma en Nanomedicina-IBIMA Plataforma BIONAND, Hospital Universitario Regional de Málaga, Unidad Clínica de Salud Mental, Av. Carlos Haya 82, 29010 Malaga, Spain; juandecara@uma.es (J.D.); antonio.lopez@ibima.eu (A.J.L.-G.); 4 Department of Cell Biology, Genetics and Physiology, Faculty of Science, University of Málaga, 29071 Malaga, Spain; 5 Biomedical Research Institute of Malaga and Platform in Nanomedicine (IBIMA-BIONAND Platform), 29590 Malaga, Spain; 6 Central Research Support Services (SCAI), University of Málaga, Campus de Teatinos s/n, 29071 Malaga, Spain; ccg@uma.es; 7 Laboratory of Chemistry of Natural Products, Department of Botany, Faculty of Natural and Oceanographic Sciences, University of Concepción, Concepción 3040000, Chile; cristianriquelme@protonmail.com (C.R.); claudiaperez@udec.cl (C.P.M.); 8 Department of Sciences and Natural Resources, Faculty of Sciences, University of Magallanes, Punta Arenas 6200000, Chile; marisel.araya@umag.cl (M.A.-R.); victor.fajardo@umag.cl (V.F.)

**Keywords:** *Bovistella utriformis*, fungal polysaccharides, wound healing, quantitative proteomics, complement cascade, zebrafish model, topical formulation, skin regeneration

## Abstract

Natural fungal polysaccharides are increasingly explored as bioactive compounds capable of orchestrating complex regenerative responses during tissue repair. This study aimed to evaluate the in vivo wound-healing efficacy and molecular mechanisms of a topical polysaccharide formulation derived from *Bovistella utriformis* (Calvatin 2%) using complementary murine, zebrafish, and proteomic approaches. Phylogenetic analysis based on ITS sequences confirmed the taxonomic identity of the Chilean specimen. In a murine full-thickness excisional wound model, Calvatin 2% significantly accelerated wound contraction and re-epithelialization compared to both saline and base-cream controls, achieving near-complete closure by day 10. Label-free quantitative proteomic analysis of wound tissue by UHPLC-HRMS identified 2432 high-confidence proteins, with 171 upregulated and 153 downregulated proteins in the Calvatin versus control comparison (*p* < 0.01). Functional enrichment revealed strong activation of innate immune response, complement activation, coagulation cascades, and acute-phase response pathways, while lipid metabolism, mitochondrial energy production, and muscle-related processes were significantly downregulated. KEGG pathway analysis further highlighted complement and coagulation cascades and neutrophil extracellular trap formation as the most prominently affected pathways. In a zebrafish laser-induced wound model, Calvatin induced early and sustained regenerative responses, reaching over 93% wound closure by 18 days post-lesion, significantly outperforming both PBS and vehicle-treated groups. Chronic oral administration of polysaccharides did not induce major hepatic inflammatory responses, supporting systemic safety. Overall, these findings indicate that *B. utriformis* polysaccharides are associated with modulation of immune- and repair-related pathways together with tissue reprogramming processes that may contribute to accelerated cutaneous regeneration, positioning Calvatin as a promising bioactive formulation for wound-healing applications.

## 1. Introduction

Fungal polysaccharides have emerged as promising multitarget biomacromolecules with significant therapeutic potential in tissue repair and regenerative medicine. Increasing evidence indicates that medicinal mushrooms modulate key molecular pathways involved in inflammatory resolution, oxidative stress mitigation, extracellular matrix remodeling, and cellular proliferation. However, the depth of mechanistic understanding varies considerably among species.

Mechanistic evidence is particularly advanced for *Hericium erinaceus*, whose secondary metabolites stimulate nerve growth factor (NGF) synthesis and activate ERK and PI3K/Akt signaling pathways, promoting neurite outgrowth and peripheral nerve regeneration [1,2]. In vivo studies further demonstrate enhanced collagen deposition, angiogenesis, and accelerated wound repair, illustrating how defined fungal metabolites can drive specific regenerative cascades [3].

Species of the genus *Pleurotus*, particularly *Pleurotus ostreatus*, have demonstrated anti-inflammatory and wound-healing effects in excisional wound models, partly attributed to β-glucans (pleuran) with immunomodulatory activity [4,5]. Likewise, *Lentinula edodes* produces lentinan and related polysaccharides that regulate NF-κB and MAPK signaling, contributing to immune modulation and oxidative stress protection [6,7]. Nevertheless, despite these advances, comprehensive proteomic characterization of treatment-specific signaling networks during in vivo wound repair remains limited for most fungal polysaccharides.

In a previous study, an extensive evaluation of the cytotoxic safety and regenerative potential of *Bovistella utriformis* polysaccharides (PsBu) was conducted using complementary in vitro and in vivo models, including human keratinocytes, zebrafish regeneration assays, and label-free quantitative proteomics [8]. This work demonstrated that PsBu is non-cytotoxic over a broad concentration range, promotes keratinocyte migration, accelerates wound closure, and modulates molecular pathways associated with tissue repair, the oxidative stress response, and cell-cycle regulation. Early proteomic signatures indicated activation of pro-healing and cytoprotective programs, supporting the translational potential of these fungal polysaccharides as natural wound-healing agents [9].

Building on these findings, the present study extends that work by elucidating the in vivo wound-healing efficacy and molecular mechanisms of PsBu in a more complex mammalian system. For this purpose, *B. utriformis* from Chile was used and identified both morphologically and molecularly. Here, a topical formulation containing *B. utriformis* polysaccharides (Calvatin 2%) was evaluated in a murine full-thickness excisional skin-wound model, and the wound tissue was subjected to UHPLC-HRMS-based quantitative proteomic profiling. This integrative approach enables characterization of treatment-specific proteomic remodeling and distinction between polysaccharide-driven effects and vehicle-related responses. It allows identification of key biological processes governing immune activation, hemostasis, inflammatory resolution, extracellular matrix dynamics, and metabolic reprogramming during skin repair.

By integrating macroscopic wound-healing assessment, zebrafish regeneration kinetics, and deep quantitative proteomic analysis, this study provides an integrative framework linking proteomic changes with organism-level tissue regeneration. Collectively, these findings further substantiate the therapeutic potential of *B. utriformis* polysaccharides and position them among mechanistically characterized fungal biomacromolecules under development for polysaccharide-based wound-healing formulations.

## 2. Results

### 2.1. Species Identification and Phylogenetic Analysis

**Figure 1:**
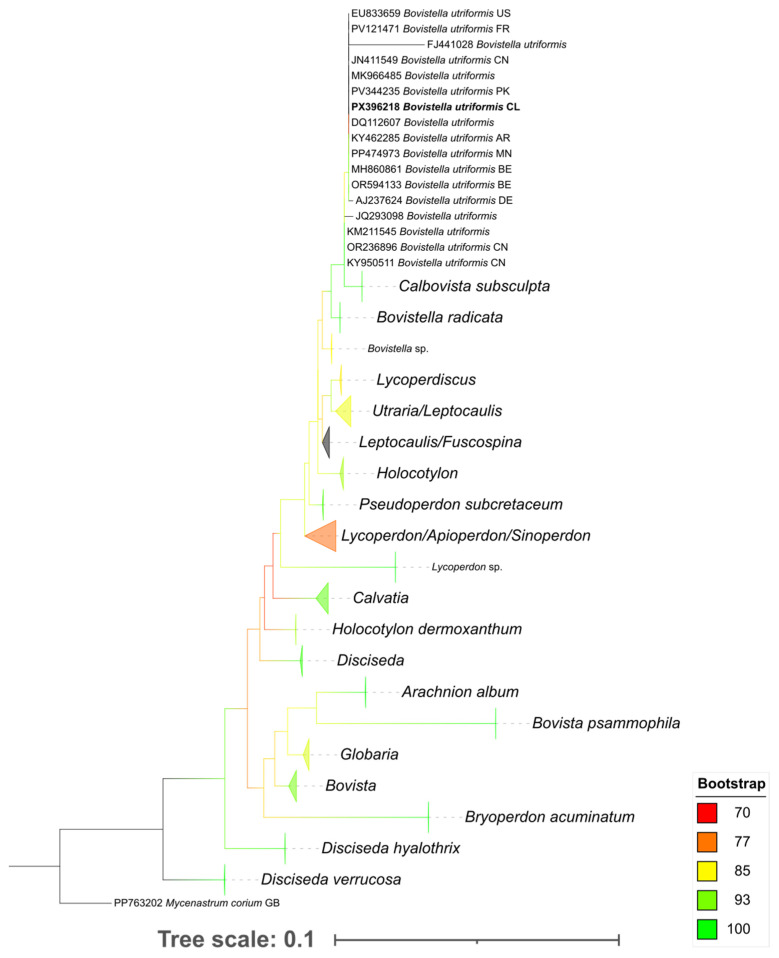
Phylogenetic relationships of Lycoperdaceae inferred from ITS region sequences using maximum likelihood analysis. Branches were considered supported by bootstrap values ≥ 70 and are represented by a color gradient from red (70) to green (100).

The family-level phylogenetic tree (Figure 1) revealed well-resolved terminal branches, with most clades supported by high bootstrap values ranging from 93 to 100, indicating a strong reliability in the grouping of closely related sequences. In contrast, some internal nodes showed lower support values (70–85), indicating some uncertainty in the deeper phylogenetic relationships among major lineages. This pattern suggests that while species- or strain-level affiliations are robustly resolved, higher-level relationships remain less certain and may require additional genetic markers or multilocus analyses to achieve greater phylogenetic resolution. In particular, *B. utriformis* from Magallanes, Chile, clusters with high bootstrap support alongside specimens from Europe, Asia, and South America.

### 2.2. In Vivo Skin Wound Healing Evaluation in Mice

**Figure 2:**
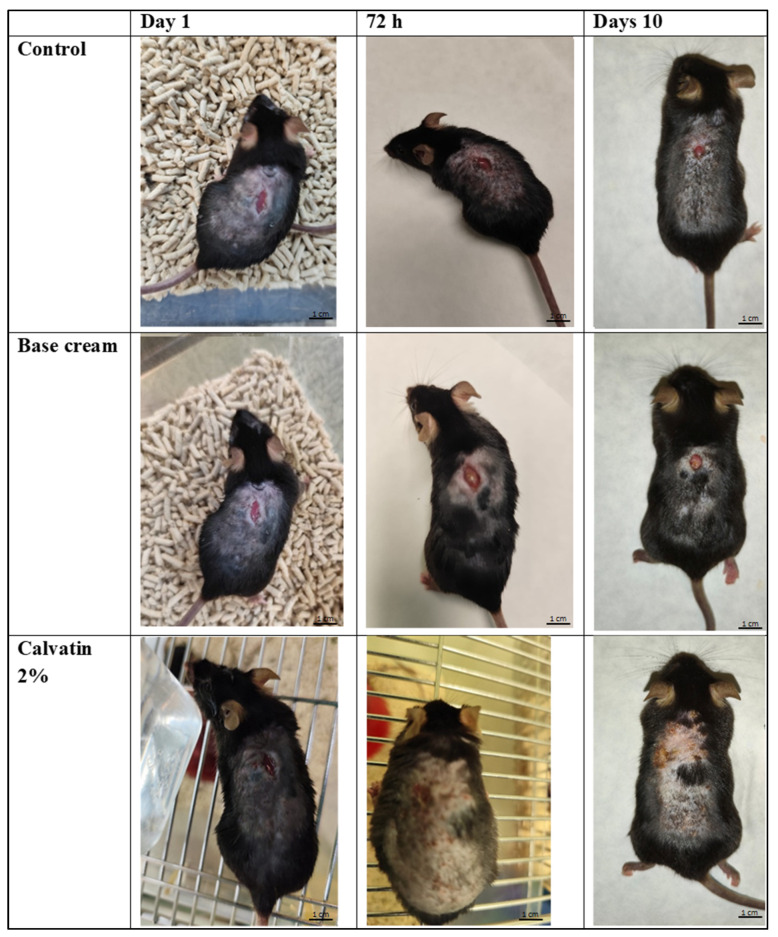
Macroscopic evaluation of wound healing in mice treated with physiological saline (Control), base cream, or Calvatin 2%. Representative images of excisional skin wounds at day 0, 72 h, and 10 days post-injury are shown for each group.

Macroscopic evaluation of wound healing progression (Figure 2) revealed significant differences among the three groups (control, base cream, and Calvatin 2%). At day 0, all animals presented comparable wound morphology and lesion size. After 72 h, the control group (treated with physiological saline) and the base cream group exhibited persistent open wounds. The inflammatory borders were prominent and wound contraction was minimal, clearly showing that tissue repair was slow. In contrast, the Calvatin 2% group showed a pronounced wound contraction, with partial closure and reduced erythematous borders, suggesting an accelerated healing response. By day 10, wounds in both the control and base cream groups remained incompletely closed, with visible open areas and clear signs of delayed epithelialization. Conversely, mice treated with Calvatin 2% displayed almost complete wound closure, with full re-epithelialization and restoration of the skin surface, leaving only a faint scar. These findings highlight the potent wound-healing efficacy of Calvatin, which markedly accelerates wound contraction and epithelial regeneration compared to the control and vehicle-treated groups. These phenotypic improvements were further explored at the molecular level through proteomic analysis of wound tissue collected at day 10. It should be noted that photographic documentation of wounds at Day 0 was not performed under a standardized imaging protocol, which precluded retrospective digital planimetry. However, wound size comparability at baseline is supported by the strict surgical standardization applied: all full-thickness wounds of identical length (1 cm) were created in a single session by the same operator, under general anesthesia, in animals of equivalent age and body weight (C57BL/6, 4–5 weeks, 24–26 g), as accepted in published murine wound healing studies [10,11]. This limitation is acknowledged and standardized planimetry will be incorporated in future studies.

### 2.3. Proteomic Profiling of Wound Tissue in a Murine Model

A total of 3228 protein groups, comprising 3866 proteins, were identified across all samples. After applying stringent filtering criteria, 2432 high-confidence proteins were retained for subsequent analyses. High-confidence proteins were defined as master proteins within their respective protein groups, identified with a false discovery rate below 1% and supported by at least two unique peptides.

Comparative analysis between experimental groups revealed a substantial number of differentially expressed proteins. In the comparison between the Calvatin group and the control group, 153 proteins were downregulated, and 171 were upregulated. The comparison between the base cream-treated group and the control group identified 47 downregulated and 27 upregulated proteins. Finally, comparison of the Calvatin group versus the base cream–treated group revealed 140 downregulated and 252 upregulated proteins.

The complete list of differentially expressed proteins is provided in Supplementary Table S1.

**Figure 3:**
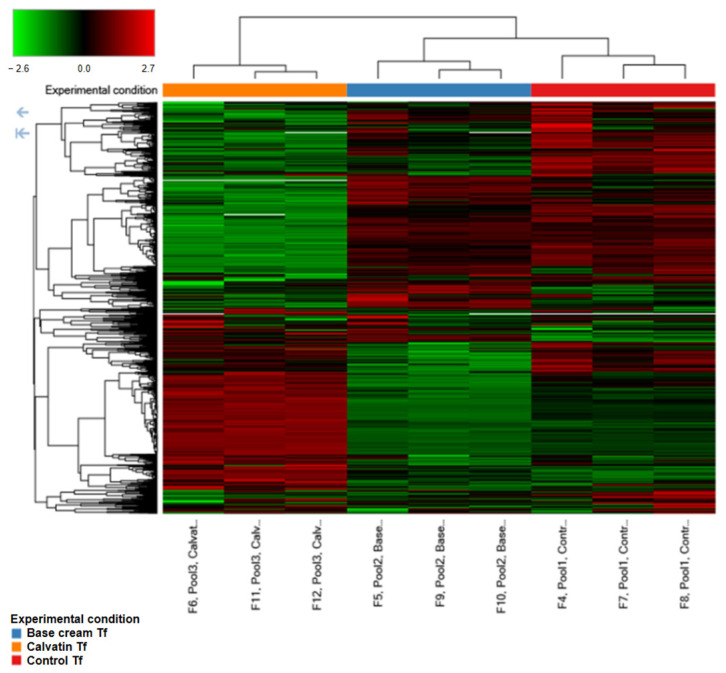
Hierarchical clustering heatmap of differentially expressed proteins across experimental groups. The heatmap represents log_2_-transformed protein abundance ratios for control, base cream-treated, and Calvatin-treated wound tissue samples. Proteins are shown in rows and samples in columns. Color intensity indicates relative expression levels, with red and blue denoting upregulation and downregulation, respectively. Clustering was performed using unsupervised hierarchical clustering, revealing a distinct proteomic profile associated with Calvatin treatment.

The hierarchical clustering heatmap of differentially expressed proteins (Figure 3) revealed a clear segregation of samples according to treatment condition. Protein expression profiles from the Calvatin group clustered distinctly from both the control and base cream–treated groups, indicating a pronounced and reproducible treatment-specific proteomic signature. In contrast, samples from the base cream–treated group showed a protein expression pattern more closely related to the control group, suggesting a limited impact of the vehicle alone on the global proteome.

The heatmap also highlighted consistent patterns of protein upregulation and downregulation across technical replicates within each experimental group, supporting the robustness and reproducibility of the proteomic measurements. Notably, the Calvatin–treated samples displayed a broader range and higher magnitude of expression changes, consistent with the higher number of differentially expressed proteins identified in pairwise comparisons.

**Figure 4:**
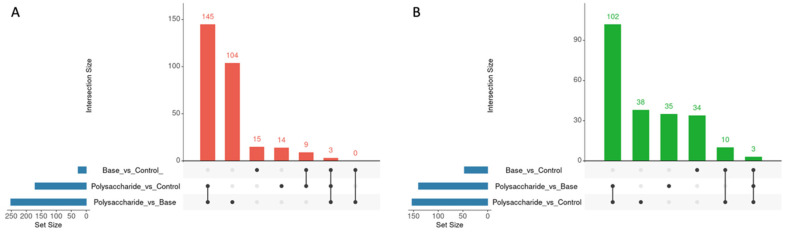
UpSet plot showing the distribution and overlap of differentially expressed proteins among the three pairwise comparisons: base cream versus control, Calvatin-treated (polysaccharide) versus control, and Calvatin versus base cream. Panel (**A**) depicts upregulated proteins, while Panel (**B**) shows downregulated proteins. Vertical bars represent the size of each intersection, and the dot matrix below indicates the combinations of comparisons contributing to each intersection. Horizontal bars on the left indicate the total number of differentially expressed proteins identified in each comparison. This representation highlights treatment-specific, shared, and vehicle-associated proteomic changes, as well as a limited subset of proteins potentially reflecting additive or synergistic effects between the base cream and Calvatin.

The UpSet plot (Figure 4) depicts the distribution and overlap of differentially expressed proteins across the three pairwise comparisons: base cream versus control, Calvatin versus control, and Calvatin versus base cream. Panel A shows upregulated proteins, whereas panel B displays downregulated proteins.

For upregulated proteins (Figure 4A), the largest intersections corresponded to proteins specifically associated with Calvatin treatment. The most prominent sets comprised 145 proteins uniquely upregulated in the polysaccharide-treated versus control comparison and 104 proteins exclusively upregulated in the polysaccharide-treated versus base cream comparison. In contrast, only 15 proteins were uniquely upregulated in the base cream versus control comparison, indicating a limited inductive effect of the vehicle alone. A smaller subset of 14 proteins was uniquely upregulated in the polysaccharide-treated versus control comparison, while 9 proteins were shared between the base cream versus control and polysaccharide-treated versus control comparisons, indicating proteins that are responsive in both treatment contexts. Within this shared set, 3 proteins were common to all three comparisons.

For downregulated proteins (Figure 4B), a similar pattern was observed. The largest group consisted of 102 proteins uniquely downregulated in the polysaccharide-treated versus control comparison, highlighting a strong suppressive effect associated with polysaccharide treatment. Additional downregulated proteins were detected exclusively in the base cream versus control (34 proteins) and polysaccharide-treated versus base cream (35 proteins) comparisons. Smaller shared subsets included 38 proteins common to polysaccharide-treated versus control and base cream versus control, 10 proteins shared between base cream versus control and polysaccharide-treated versus base cream, and 3 proteins common to all three comparisons.

Overall, the UpSet analysis indicates that the largest proportion of proteomic changes is specifically associated with Calvatin treatment, as evidenced by the high number of proteins uniquely deregulated in comparisons involving the polysaccharide-treated groups. These results highlight a robust and treatment-specific remodeling of the wound proteome, clearly exceeding the magnitude of changes observed in the base cream–treated condition and underscoring the dominant contribution of the polysaccharide formulation to the observed protein expression profiles.

**Figure 5:**
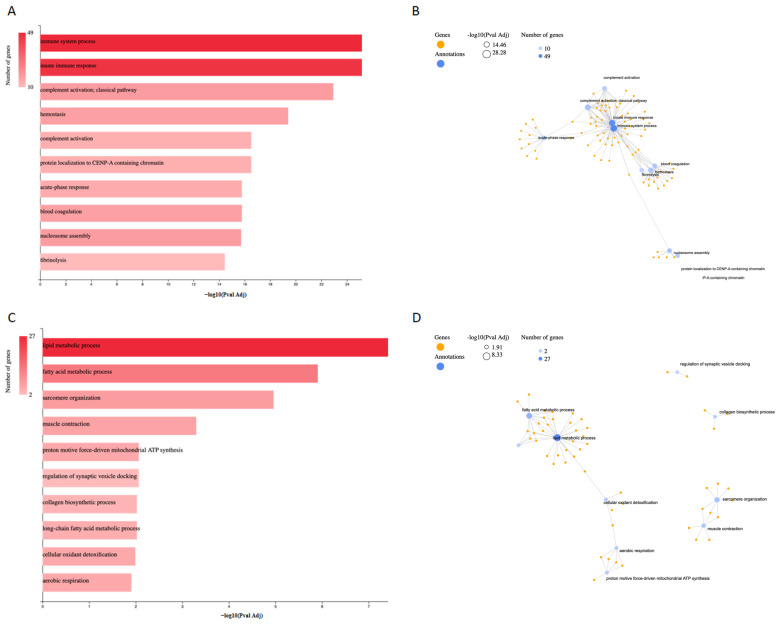
Functional enrichment analysis of differentially expressed proteins in wound tissue following treatment with Calvatin using GeneCodis. (**A**) Gene Ontology (GO) biological process enrichment of upregulated proteins, represented as a bar plot showing the most significantly enriched terms ranked by −log_10_ adjusted *p*-value; bar color intensity reflects the number of associated genes. (**B**) GO biological process network of upregulated proteins, illustrating functional relationships among enriched immune-, coagulation-, and inflammation-related processes; node size corresponds to the number of genes and node color intensity represents −log_10_ adjusted *p*-value. (**C**) GO biological process enrichment of downregulated proteins, highlighting pathways related to lipid metabolism, energy production, and muscle function. (**D**) GO biological process network of downregulated proteins, showing functional clustering of metabolic, mitochondrial, and contractile processes. Enrichment analyses were performed using GeneCodis, and statistical significance was determined using adjusted *p*-values.

Functional enrichment analysis of proteins differentially expressed in wound tissue following treatment with Calvatin revealed distinct functional signatures for upregulated and downregulated protein sets (Figure 5).

For upregulated proteins, Gene Ontology (GO) biological process enrichment analysis (Figure 5A) showed a strong overrepresentation of processes related to the immune response. The most significantly enriched terms included immune system process and innate immune response, which displayed the highest statistical significance and involved the largest number of proteins. In addition, pathways associated with complement activation, including the classical complement pathway, were prominently enriched. Processes related to hemostasis, blood coagulation, and fibrinolysis were also significantly overrepresented, indicating enrichment of coagulation-related processes in response to polysaccharide treatment. Furthermore, enrichment of terms such as acute-phase response is consistent with the involvement of early inflammatory and host-defense-related processes within the wound environment. Beyond immune and inflammatory pathways, enrichment was also observed for processes linked to chromatin organization, including nucleosome assembly and protein localization to CENP-A–containing chromatin, suggesting alterations in nuclear organization and transcriptional regulation. The GO biological process network analysis (Figure 5B) further illustrates the functional interconnections among these enriched terms, highlighting immune-related processes as a central hub closely linked to coagulation and acute-phase response pathways.

In contrast, functional enrichment analysis of downregulated proteins revealed a marked enrichment of biological processes associated with lipid and energy metabolism (Figure 5C). The most significantly enriched GO biological process was lipid metabolic process, which involved the highest number of downregulated proteins and exhibited the strongest statistical significance. Closely related terms, including fatty acid metabolic process and long-chain fatty acid metabolic process, were also prominently represented, indicating a coordinated downregulation of lipid metabolism–associated pathways. In addition, several downregulated proteins were associated with mitochondrial energy production, as reflected by the enrichment of aerobic respiration and proton motive force–driven mitochondrial ATP synthesis, suggesting a reduction in oxidative energy metabolism following polysaccharide treatment. Processes related to muscle function and cytoskeletal organization, such as sarcomere organization and muscle contraction, were also significantly enriched among downregulated proteins. Moreover, enrichment of the collagen biosynthetic process indicates modulation of extracellular matrix–related pathways at this stage of wound healing. The GO network representation of downregulated processes (Figure 5D) revealed functional clustering, with lipid and fatty acid metabolism forming a central hub connected to oxidative metabolism and cellular detoxification pathways.

Overall, these results indicate that polysaccharide treatment is associated with a marked shift in wound tissue protein expression toward immune- and host-defense–related processes, concomitant with a suppression of metabolic, contractile, and energy-demanding pathways in wound tissue.

In addition to Gene Ontology-based enrichment, pathway analysis using the Kyoto Encyclopedia of Genes and Genomes (KEGG) was performed considering the complete set of differentially expressed proteins. This analysis revealed a significant enrichment of pathways related to immune defense and inflammatory responses, with Complement and coagulation cascades emerging as the most prominently affected pathways, comprising 41 deregulated proteins. A substantial proportion of these proteins mapped to key components of both the complement system and the coagulation cascade, including factors involved in complement activation, amplification, regulation, and downstream effector functions, as well as proteins participating in fibrin formation, fibrinolysis, and platelet-related processes (Supplementary Table S2; Supplementary Figure S1).

Moreover, Neutrophil extracellular trap (NET) formation was identified as another highly enriched pathway, encompassing 40 deregulated proteins. Proteins associated with this pathway included components involved in neutrophil activation, reactive oxygen species generation, chromatin remodeling, histone modification, and granule-derived effector mechanisms, reflecting extensive modulation of molecular processes underlying NET formation (Supplementary Table S3; Supplementary Figure S2).

Taken together, the functional enrichment analyses based on Gene Ontology and KEGG pathways reveal a consistent, coordinated remodeling of the wound proteome following Calvatin treatment. The upregulated protein set was predominantly associated with immune- and inflammation-related biological processes, including innate immune response, complement activation, coagulation, and acute-phase response, whereas downregulated proteins were mainly linked to metabolic pathways, mitochondrial energy production, muscle-related processes, and extracellular matrix–related processes, including collagen biosynthesis. KEGG pathway analysis further reinforced these findings by identifying Complement and coagulation cascades and Neutrophil extracellular trap formation as the most prominently affected pathways, encompassing a large proportion of the deregulated proteins. Overall, these results indicate that polysaccharide treatment induces a marked shift in wound tissue protein expression toward immune and host-defense–related processes, concomitant with a suppression of metabolic and energy-demanding pathways at this stage of wound healing.

To validate these findings in an alternative regenerative model, we employed zebrafish, where wound repair relies primarily on epithelial migration and immune coordination rather than tissue contraction, providing complementary evidence for PsBu’s therapeutic mechanisms.

### 2.4. In Vivo Skin Wound Healing Evaluation in a Zebrafish Model

**Figure 6:**
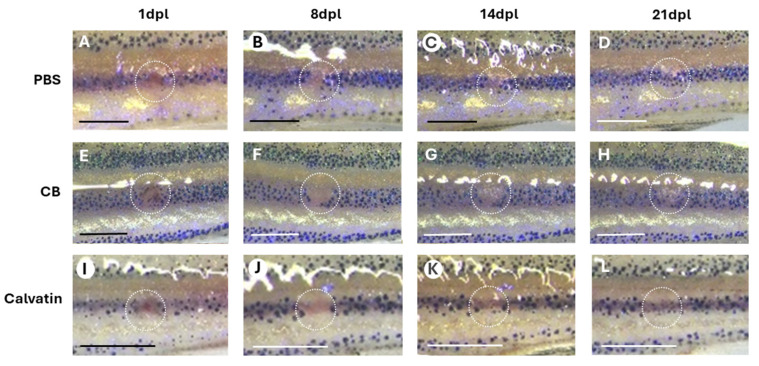
Representative images of zebrafish individuals subjected to different treatments (PBS, CB (Cream base) and calvatin). Panels (**A**–**D**), (**E**–**H**) and (**I**–**L**) show the wound of the same individual at 1 dpl (1 day post-laser), 8 dpl, 14 dpl, and 21 dpl, respectively, for each treatment. The wound area at the different time points is indicated by a white circle. Scale bar: 1 mm.

The laser-induced wound model in zebrafish revealed a clear divergence in wound-closure dynamics among treatments, with Calvatin displaying the most pronounced pro-healing effect (Figure 6).

The temporal evolution of the healing percentage showed that Calvatin initiated tissue regeneration earlier than both controls: reductions in wound area were already evident at 4–6 dpl, and by 8 dpl, most individuals exhibited advanced closure, reaching 38–88% depending on the sample. This rapid progression was reflected in the group means, which increased sharply from 6 to 14 dpl, attaining ~45% at 14 dpl and rising to 93.26% by 18 dpl, indicating that nearly all Calvatin-treated organisms had achieved—or were close to achieving complete re-epithelialization. From 18 to 28 dpl, wound closure remained stably high (93–95%), confirming the robustness and durability of the regenerative response. In contrast, the PBS group followed the expected physiological trajectory, characterized by initial fluctuations and episodes of transient wound enlargement at 4–6 dpl, with substantial healing only becoming evident after 14 dpl, full closure generally occurred between 18 and 25 dpl, and the mean healing rate remained consistently inferior to that of Calvatin (e.g., 55.62% at 14 dpl and 89.27% at 18 dpl). The cream base (CB) group exhibited the most impaired regeneration profile: strong negative values from 1 to 14 dpl indicated a marked increase in wound size before any repair was initiated, suggesting irritation or an absence of bioactive support. Only a subset of individuals reached later healing stages, and closure remained incomplete in many cases, with the average reaching merely 46.08% at 18 dpl.

**Figure 7:**
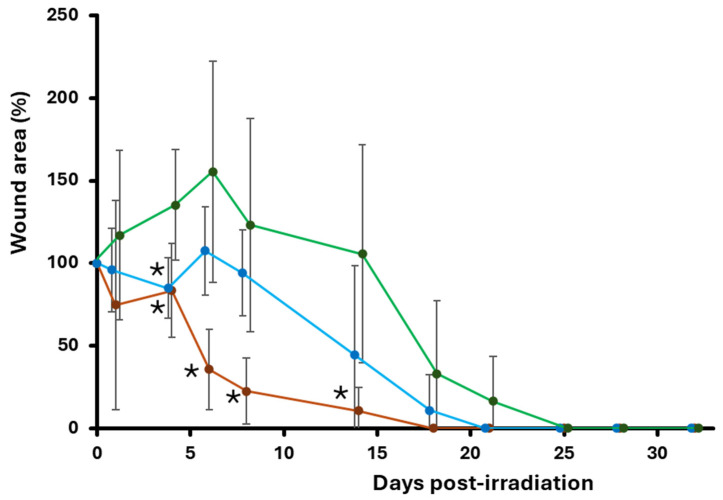
Graphical representation of the percentage of initial wound area measured on different days post-laser irradiation. The brown, blue and green lines connect the mean values for calvatin, PBS and CB, respectively. Vertical lines are standard deviations. Significant statistical differences between means at each time point are only found when comparing with CB data (asterisks; *p* < 0.05; post hoc Fisher’s Least Significant Difference (LSD) test of the One Way-ANOVA).

Collectively, these kinetic profiles demonstrate a clear separation between treatments. In principle, CB treatment markedly delays the onset of repair and compromises healing efficiency. PBS kinetic reflects natural but slow regeneration, only significantly different from CB control at early stages (asterisk at 4 dpi in Figure 7). Finally, Calvatin induces an early fluctuation in wound area and a steep, sustained healing slope statistically different from CB data (asterisks in Figure 7) but not PBS.

PBS reflects natural but slow regeneration, and CB markedly delays the onset of repair and compromises healing efficiency (Figure 7). To evaluate systemic safety for potential therapeutic applications, we analyzed hepatic inflammatory markers following chronic oral PsBu administration.

### 2.5. Analysis of Inflammation- and Injury-Related Gene Expression in the Liver

**Figure 8:**
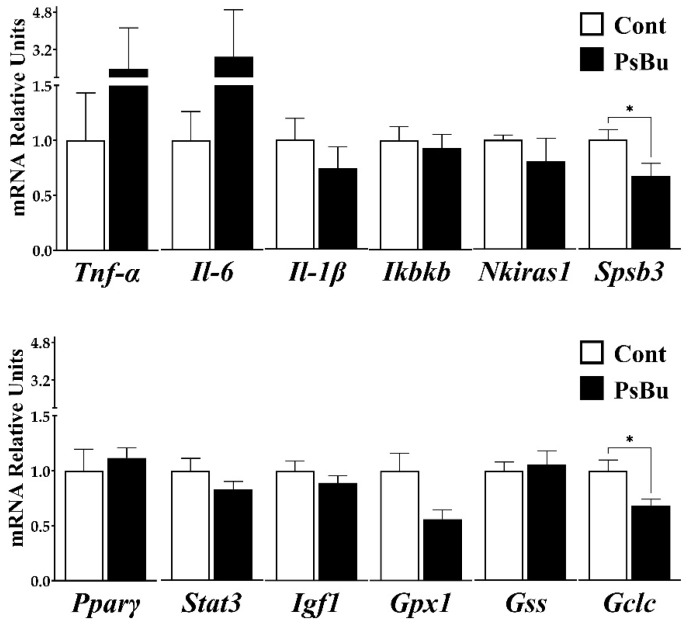
Hepatic gene expression profile of inflammation- and liver injury-related markers in control and PsBu-treated mice. Relative mRNA levels of Tnf-α, Il-6, Il-1β, Ikbkb, Nkiras1 and Spsb3 (upper panel) and Pparγ, Stat3, Igf1, Gpx1, Gss and Gclc (lower panel) were quantified in liver samples from mice chronically (40 days) treated with PsBu or vehicle (Cont) (*n* = 10). Data are expressed as relative mRNA units (mean ± SEM) analyzed by Student’s *t*-test (* *p* < 0.05 PsBu vs. Cont).

Chronic oral administration of PsBu did not significantly modify hepatic mRNA levels of the pro-inflammatory cytokines Tnf-α, Il-6, and Il-1β compared with the control group, although a mild, non-significant increase was observed for Tnf-α and Il-6 (Figure 8). PsBu treatment also failed to alter the expression of the upstream inflammatory regulators, Inhibitor of nuclear factor kappa B kinase subunit beta (Ikbkb) and NF-κB inhibitor interacting Ras-like protein 1 (Nkiras1). In contrast, PsBu significantly reduced hepatic Spsb3 mRNA levels compared with controls (*p* < 0.05). Likewise, no significant changes were detected in the expression of Pparγ, Stat3, Igf1, Gpx1, or Gss. In contrast, Gclc mRNA was significantly decreased in the PsBu-treated group (*p* < 0.05).

## 3. Discussion

Building on previous findings, the present in vivo study was designed as a direct extension of our earlier work on polysaccharides extracted from *B. utriformis* (PsBu), which demonstrated their physicochemical stability, biocompatibility, and strong pro-regenerative activity in vitro [8]. Thermogravimetric analysis revealed that PsBu exhibits high thermal stability, maintaining structural integrity up to approximately 300 °C, supporting its suitability for pharmaceutical and biomedical formulations. Functionally, PsBu significantly enhanced keratinocyte migration and scratch wound closure in HaCaT cells in a dose-dependent manner, without inducing apoptosis or cell-cycle arrest, indicating a safe pro-regenerative profile. Proteomic analyses further revealed coordinated modulation of proteins involved in cell migration (VASP, FHL2), extracellular matrix remodeling (plasminogen), mitotic surveillance (BUB1), and nucleocytoplasmic trafficking, suggesting a tightly regulated regenerative and immunomodulatory response rather than uncontrolled proliferation. These molecular and cellular effects were corroborated in a zebrafish fin regeneration model, where PsBu accelerated tissue regeneration at concentrations lower than those required in vitro, highlighting its bioactivity in a whole-organism context. Collectively, these results provided a strong mechanistic and biological rationale for evaluating PsBu in mammalian excisional wound models and regenerative zebrafish skin injury, leading to the current comprehensive in vivo assessment of Calvatin 2% topical cream.

The macroscopic evaluation of the skin wounds in C57BL/6 mice demonstrated that topical treatment with Calvatin 2% cream markedly accelerated the wound-healing process compared to both control and base-cream groups. While control and vehicle-treated animals showed persistent open wounds, inflammatory borders, and delayed epithelialization at 72 h and day 10, Calvatin-treated mice exhibited rapid wound contraction, reduced erythema, and nearly complete re-epithelialization by day 10, leaving only minimal scarring. These findings suggest that Calvatin may enhance both the early and late phases of wound repair, including inflammation resolution, tissue contraction, and epidermal regeneration. Such effects are consistent with previous studies reporting that fungal polysaccharides, particularly β-glucans and heteropolysaccharides, significantly promote wound closure in murine models by stimulating fibroblast proliferation, myofibroblast differentiation, and extracellular matrix remodeling [10,11]. Early wound contraction observed at 72 h suggests activation of fibroblast-driven mechanisms, which are critical for reducing wound size and preparing the wound bed for re-epithelialization [12]. Moreover, the reduced erythematous borders in the Calvatin-treated group indicate a modulation of the inflammatory response, a key determinant of efficient healing, as excessive or prolonged inflammation is known to impair tissue repair. Mushroom-derived polysaccharides have been widely reported to exert immunomodulatory effects by regulating macrophage activity and cytokine production, including downregulation of pro-inflammatory mediators such as TNF-α and IL-6, while promoting growth factors essential for tissue regeneration [13]. The near-complete epithelial coverage observed at day 10 further suggests enhanced keratinocyte migration and proliferation, processes previously reported for polysaccharides isolated from *Tremella fuciformis* and *Lentinus edodes*, which stimulate epidermal regeneration through growth factor signaling pathways [14]. Importantly, the lack of significant improvement in the base cream group confirms that the observed biological effects are attributable to the PsBu itself rather than to the vehicle’s occlusive or moisturizing properties.

To further complement these findings, we performed a proteomic analysis to characterize the molecular effects of Calvatin on wound tissue, distinguishing treatment-specific changes from those attributable to the base cream. Our results demonstrate that polysaccharide treatment induces a marked and coordinated remodeling of the wound proteome, which appeared to exceed the relatively modest effects observed for the vehicle alone. A central finding was the upregulation of proteins associated with immune and inflammatory responses in polysaccharide-treated wounds. Functional enrichment analyses revealed significant enrichment of pathways related to innate immune response, complement, coagulation, and acute-phase response. These biological processes are known to play key roles during the early stages of wound healing, contributing to host defense, hemostasis, and the initiation of tissue repair. Importantly, the absence of macroscopic signs of persistent inflammation at later time points suggests that the observed proteomic signature is compatible with a regulated wound-response profile rather than overtly prolonged inflammation. Consistently, KEGG pathway analysis identified complement and coagulation cascades as the most prominently affected pathways, highlighting extensive modulation of proteins related to immune and hemostatic pathways following polysaccharide treatment. In addition, enrichment of the Neutrophil Extracellular Trap (NET) formation pathway suggests the involvement of proteins associated with neutrophil-related defense pathways within the wound microenvironment. NET-related proteins involved in chromatin remodeling, oxidative processes, and inflammatory signaling were deregulated, supporting the presence of a proteomic profile compatible with innate immune-related responses in treated wounds. The concurrent enrichment of chromatin organization–related processes further suggests broader cellular reprogramming associated with immune activation. In contrast, downregulated proteins were predominantly associated with lipid metabolism, mitochondrial energy production, muscle-related processes, and extracellular matrix–related pathways, including collagen biosynthesis. This coordinated suppression of metabolic and energy-demanding processes may reflect a transient metabolic reprogramming of wound tissue, whereby cellular resources are redirected towards immune defense and early repair mechanisms at the analyzed stage of healing. Overall, the combined GO and KEGG analyses reveal a clear dichotomy in the proteomic response to Calvatin, characterized by activation of immune and hemostatic pathways alongside suppression of metabolic and contractile functions. These findings suggest that polysaccharide treatment is associated with a wound microenvironment characterized by immune-related proteomic remodeling and functional reorganization.

An apparently unexpected observation was the enrichment of immune-related, complement, and coagulation pathways at day 10 post-injury, a time point typically associated with the resolution phase of wound healing. While these pathways are classically linked to early inflammatory responses, increasing evidence indicates that several components of the complement and coagulation systems also play important roles during later stages of tissue repair, including debris clearance, modulation of angiogenesis, and orchestration of tissue remodeling [15,16,17,18].

In this context, the observed proteomic profile may not reflect persistent or detrimental inflammation, but rather a regulated and functionally relevant involvement of immune-related pathways contributing to ongoing tissue remodeling and repair. This interpretation is consistent with the macroscopic evidence of efficient wound closure and the absence of visible signs of chronic inflammation in treated animals.

Nevertheless, the lack of proteomic data at earlier time points (e.g., day 1 or day 3 post-injury) limits our ability to define the temporal dynamics of these pathways and to distinguish between early activation and prolonged signaling. Future studies incorporating longitudinal sampling will be necessary to resolve the temporal sequence of molecular events underlying PsBu-mediated wound healing.

However, these interpretations should be made with caution. Although the proteomic analysis revealed enrichment of immune-related pathways, the present study did not include direct assessment of immune cell activation, macrophage polarization, cytokine production, or other functional immunological readouts in wound tissue. Therefore, the proposed involvement of immune-related mechanisms should be considered associative rather than demonstrative and will require dedicated validation in future studies. In addition, it is important to note that the proteomic analysis was performed on pooled wound tissue samples at a single time point, which precluded direct correlation analyses between individual protein expression levels and wound-healing outcomes. Therefore, although the observed proteomic changes are consistent with the improved healing phenotype, a quantitative correlation between protein abundance and wound closure could not be established in the present study.

The lack of major changes in the hepatic expression of classical pro-inflammatory cytokines such as Tnf-α, Il-6 and Il-1β suggests that chronic administration of the fungal polysaccharide extract does not elicit an overt inflammatory response in the liver under the present experimental conditions. The absence of effects on Ikbkb and Nkiras1 further indicates that upstream components of the NF-κB pathway remain largely unaffected, supporting the notion that PsBu is not strongly pro-inflammatory at the hepatic level. Interestingly, the significant downregulation of Spsb3 may indicate a more subtle modulation of cytokine signaling, as SPSB proteins are involved in regulating receptor-mediated pathways; this effect could contribute to fine-tuning inflammatory responses rather than robust activation.

On the other hand, the absence of changes in genes related to metabolism and antioxidant defense (Pparγ, Stat3, Igf1, Gpx1, Gss) suggests that PsBu does not markedly disturb hepatocellular homeostasis. However, reduced Gclc expression may indicate a reduced capacity for glutathione synthesis, rendering hepatocytes more susceptible to oxidative or toxic insults under additional stress. Together, these findings point to a profile in which the polysaccharide extract does not provoke obvious liver inflammation or damage by itself but may subtly modulate cytokine signaling and redox-related pathways.

Wound repair in zebrafish relies on rapid keratinocyte migration, coordinated inflammatory resolution, and efficient epithelial remodeling rather than contraction, making it a powerful system to study true regenerative processes [19,20]. Our results in the zebrafish laser-induced cutaneous wound model support the promotion of wound healing by topical application of Calvatin, consistent with a previous report on a similar effect exerted by *Spirulina maxima* polysaccharides [21]. Calvatin significantly enhances tissue regeneration compared to both PBS and cream base controls. Calvatin-treated fish exhibited an early onset of wound closure at 4–6 days post-laser (dpl), something not found in the PBS group, which showed a transient wound enlargement and delayed closure, or in the cream base group, which showed pronounced negative healing values during the first two weeks, suggesting irritation or lack of bioactive support. We propose that this impaired regeneration may be attributable, at least in part, to the presence of benzyl alcohol in the cream base formulation (Supplementary Material S2). Although benzyl alcohol is widely used as a cosmetic preservative, zebrafish (*Danio rerio*) display heightened sensitivity to this compound [22], demonstrating significant developmental toxicity at micromolar concentrations, including cardiovascular defects and disruption of normal tissue development. We propose that even low concentrations applied topically to wounded skin may provoke a local irritant response in this sensitive model organism, transiently impairing epithelial regeneration. Importantly, this delay was not observed in Calvatin-treated fish despite sharing the same cream base, suggesting that the bioactive PsBu fraction counteracts the vehicle effect through its anti-inflammatory and cytoprotective properties [8]. Quantitatively, Calvatin induced a steep and sustained healing slope, with mean wound closure reaching approximately 45% at 14 dpl and exceeding 93% by 18 dpl, indicative of robust and durable re-epithelialization. These dynamics of pigment restoration are similar to those observed by other authors using algal polysaccharides [21]. The delayed and incomplete healing observed in the cream base group further confirms that the regenerative effect is attributable to the PsBu itself rather than to physical or occlusive properties of the formulation. These findings are consistent with previous reports demonstrating that bioactive polysaccharides, especially those derived from fungi, promote zebrafish skin regeneration by modulating innate immune responses, limiting excessive inflammation, and enhancing epithelial cell migration and proliferation [21]. The sustained high closure rates observed after 18 dpl in Calvatin-treated fish suggest not only accelerated initiation of repair but also long-term stabilization of the regenerated tissue, reinforcing the potential of PsBu as an effective pro-regenerative agent. Overall, the zebrafish data further provide strong in vivo evidence that Calvatin enhances both the onset and efficiency of cutaneous wound healing, supporting its translational relevance for topical regenerative therapies.

While the present findings provide integrated mechanistic and functional insights into PsBu-mediated wound repair, additional studies incorporating detailed histological analyses, local cytokine profiling, and long-term remodeling assessments would further refine the understanding of its immunomodulatory and regenerative mechanisms.

Taken together, the macroscopic, proteomic, and zebrafish regeneration data converge to show that PsBU exerts a robust and multifaceted pro-healing effect across complementary in vivo models and biological scales. In mice, Calvatin 2% cream accelerated wound contraction and re-epithelialization, indicating efficient coordination of inflammatory resolution, fibroblast activation, and epidermal regeneration. Proteomic profiling revealed a treatment-specific molecular signature that is consistent with the observed macroscopic improvements in wound healing. The convergence between accelerated wound closure and the enrichment of proteins related to immune response, hemostasis, and tissue remodeling supports a biologically coherent association between proteomic remodeling and the healing phenotype, although a direct quantitative correlation could not be established in the present study. Specifically, the enrichment of proteins related to innate immune response, complement, coagulation, and acute-phase pathways suggests that Calvatin treatment is associated with molecular features typically linked to early wound-response programs. The concurrent enrichment of neutrophil extracellular trap–related processes further supports the involvement of wound-defense–related pathways. The downregulation of metabolic, mitochondrial, muscle-related, and collagen biosynthesis pathways likely reflects a transient metabolic shift in wounded tissue, potentially prioritizing wound-response and early repair processes over energy-intensive structural remodeling at the analyzed time point. Importantly, the zebrafish laser-induced wound model independently confirmed the biological relevance of these effects in a regeneration-dominant system, revealing that Calvatin induces an early fluctuating stage in healing kinetics followed by rapid, sustained, and near-complete re-epithelialization, in contrast to the slower physiological repair observed in PBS-treated fish and the impaired regeneration seen with the cream base. Because zebrafish wound repair relies primarily on epithelial cell migration and coordinated tissue responses rather than tissue contraction [21], these findings support the biological relevance of PsBu-associated regenerative effects across complementary models. Collectively, the consistency of outcomes across murine excisional wounds, molecular proteomic signatures, and zebrafish regenerative kinetics provides convergent evidence that Calvatin is associated with a wound microenvironment favorable to efficient healing, positioning PsBu as a promising bioactive agent for the development of effective and biologically grounded topical regenerative therapies.

## 4. Materials and Methods

### 4.1. Biological Material

The biological material and polysaccharide extraction protocol applied in the present study were identical to those previously reported in [8,23]. Fruiting bodies of *Bovistella utriformis* (syn. *Calvatia utriformis*) were collected in the Magallanes Region, southern Chile (54°04′00.8″ S, 68°57′27.0″ W). Immediately after collection, specimens were frozen, subsequently lyophilized, and stored under controlled conditions (dry, dark environment at −20 °C, protected from moisture and light, and sealed in vacuum bags to prevent exposure to air and contamination) before transport for polysaccharide extraction and biological analyses.

Polysaccharides were extracted according to the method described by Abdala et al. (2011) [24], as consistently applied in Maaloul et al. (2026, 2025) [8,23], to obtain the polysaccharide fraction designated PsBu. Briefly, dried fungal biomass was subjected to three successive treatments with absolute ethanol to remove pigments and low-molecular-weight compounds. The residue was then suspended in distilled water (1:10, *w*/*v*) and boiled for 1 h under continuous stirring. After centrifugation, phenolic compounds were eliminated using polyvinylpolypyrrolidone (PVPP), and polysaccharides were precipitated by the addition of cold absolute ethanol. The resulting precipitate was collected by centrifugation, frozen at −80 °C, and lyophilized. This standardized extraction protocol was maintained across all three studies to ensure methodological reproducibility and allow direct comparison of the biological results.

### 4.2. DNA Extraction, PCR Amplification, Sequencing

Genomic DNA was extracted from lyophilized fungal tissue using the GeneJET™ Genomic DNA Purification Kit (Thermo Scientific, Waltham, MA, USA), following the yeast protocol with modifications to enhance cell wall disruption. Samples were incubated either with Yeast Lysis Buffer supplemented with lysozyme (20 mg/mL) or with Yeast Cell Lysis Solution 2 (Biosearch Technologies, Petaluma, CA, USA). DNA purification was performed according to the kit instructions, and elution was done in 80 µL of Elution Buffer. DNA quality was verified by 1% agarose gels.

Four gene regions were targeted for PCR amplification: ITS (ITS1/ITS4), EF1-α (EF1-728F/EF1-986R), and two β-tubulin regions (Bt1a/Bt1b and Bt2a/Bt2b). PCR reactions were prepared in 25 µL using 2 µL of template DNA, and cycling conditions were optimized for each marker. Amplification products were visualized on 1% agarose gels stained with GreenSafe. PCR products were enzymatically purified (ExoStart) and bidirectionally sequenced (Sanger method) at the Genotyping Service (SCAI, University of Málaga, Spain). Chromatograms were inspected with Chromas. Among the amplified loci, only ITS yielded high-quality sequences suitable for phylogenetic analysis.

### 4.3. Phylogenetic Analysis

For phylogenetic reconstruction, a global dataset of 1505 full-length ITS sequences of Lycoperdaceae species from diverse geographic origins was compiled from GenBank [25]. The dataset was complemented with a newly generated sequence of *B. utriformis* from Magallanes, Chile (GenBank accession number: PX396218; Dataset 1, Supplementary Material S1). *Mycenastrum corium* was used as an outgroup following [26]. Chromatograms were edited, and consensus sequences were generated using CodonCode Aligner v12.0.1 [27]. Multiple sequence alignment was performed with MAFFT v7.490 using the E-INS-i strategy [28], and alignments were manually checked in AliView v1.3 [29]. Misannotated, incomplete, low-quality, or unalignable sequences were removed, and ambiguous regions were filtered with the -automated1 option of trimAl v1.2 [30]. Sequence renaming was performed with TBtools-II v2.154 [31].

The best-fitting substitution model was selected with ModelTest-NG v0.1.7 [32] under the corrected Akaike information criterion (AICC). Phylogenetic inference was conducted with RAxML-NG v1.1.0 [33] using the maximum likelihood method with 1000 bootstrap replicates [34], integrated via raxmlGUI v2.0.10 [35]. The resulting tree was visualized and annotated in iTOL v7 [36] and finalized in Inkscape v1.4.2 [37]. All data supporting this analysis are available in Supplementary Material S1).

### 4.4. Formulation of Wound-Healing Cream

A topical cream formulation containing the purified polysaccharide extract from *B. utriformis* (PsBu) was prepared using a certified organic commercial cream base (Crema base, Cremas Caseras, Spain). The base composition (INCI) includes: *Aloe barbadensis* leaf juice*, Caprylic/capric triglyceride, Glycerin**, Cetyl alcohol, Cetearyl olivate, Butyrospermum parkii butter*, Sorbitan olivate, Glyceryl caprylate, Tocopherol, Xanthan gum, Glyceryl undecylenate, Phytic acid, Aqua, and Benzyl alcohol (*ingredients from organic farming; **processed from organic ingredients; 81% of total ingredients from organic farming, 99% of natural origin). The technical data sheet of the cream base is provided in the Supplementary Material S2.

The polysaccharide extract (PsBu) was incorporated into the base at a final concentration of 2% (*w*/*w*). An antibiotic (ATB, 0.5% *w*/*w*) was added to prevent microbial contamination. The mixture was homogenized under sterile conditions until the polysaccharide was completely dispersed. The final formulation, hereafter referred to as Calvatin 2%, was stored at room temperature in hermetically sealed sterile containers until further use in wound-healing assays.

### 4.5. In Vivo Skin Wound Model

#### 4.5.1. Animals and Ethics Statement

The study was carried out in the Animals Experimentation Center of the University of Malaga, and a total of 10 mice (*n* = 10) were used in this study, performed on 4 to 5-week-old C57BL/6 mice (Charles River Laboratories, Barcelona, Spain) weighing 24-26 g at the start of the study. Mice were housed in appropriate cages for a maximum of five individuals maintained under a standard 12 h light-dark cycle in a room under temperature/humidity control with ad libitum access to water and food. Animal handling and care adhered to the European Communities Council Directives 2010/63/EU (Regulation EC 86/609/ECC, 24 November 1986) and Spanish National and Regional Guidelines for Animal Experimentation (Real Decreto 53/2013). All experimental protocols received approval from the Ethical Committee for Animal Research at the University of Malaga (CEUMA #43-2021-A and·#9-2025-A) and were conducted in accordance with the ARRIVE guidelines (Animal Research: Reporting of in vivo Experiments) [38].

#### 4.5.2. Experimental Skin Wound Design and Treatment

Twenty mice were anesthetized using inhalation anesthesia with isoflurane (Isofluorin, Vetpharma Animal Health SL, Barcelona, Spain), and the dorsal region was mechanically shaved. Full-thickness excisional wounds (1 cm in length) were created using sterile surgical scissors. Then, the animals were divided into three randomized experimental groups: PsBu group treated with *B. utriformis* polysaccharide-extract topical cream (Calvatin), vehicle group treated with the topical base-cream and control group treated with physiological saline. Mice were topically treated daily with 1 mL per wound of both PsBu and Control for 10 days. Finally, the animals were euthanized with an intraperitoneal (ip) administration of sodium pentobarbital (Dolethal, Vetoquinol, Alcobendas, Spain, #QN51AA01) overdose (50 mg/kg body weight). Subsequently, wound lesions were removed with adjacent healthy skin.

#### 4.5.3. Chronic Oral PsBu Treatment and Liver Tissue Collection for Molecular Analysis

PsBu was dissolved in 2% (*w*/*v*) methylcellulose of 400 cP viscosity in water (#M0262, Sigma-Aldrich, St. Louis, MI, USA) and previously diluted in deionized water to obtain a 0.5% v/v solution. Mice (*n* = 10) received chronic treatment for 40 days with the PsBu solution, which was administered orally at a dose of 200 mg/kg in a volume of 10 mL/kg BW. The control group (Cont) was treated in the same manner but received the vehicle solution instead.

On the last day of treatment, two hours after administration of PsBu or vehicle, the animals were anesthetized ip with 50 mg/kg body weight sodium pentobarbital and sacrificed by exsanguination. The liver was dissected and immediately frozen at −80 °C for subsequent gene expression analysis

### 4.6. Proteomic Analysis

Wound tissue samples were collected 10 days after wound induction and processed for proteomic analysis. Three pooled samples were analyzed, each pool consisting of wound tissue obtained from six mice. The experimental groups included a control pool (no treatment applied to the wound), a base cream-treated pool, and a pool treated with PsBu cream Calvatin 2%. Each pooled sample was analyzed in triplicate, corresponding to three technical replicates.

Proteomic profiling was performed using ultra-high-performance liquid chromatography coupled to high-resolution mass spectrometry (UHPLC-HRMS) to assess the effects of Calvatin on global protein expression patterns in wound tissue.

Wound tissue samples were homogenized in RIPA buffer (Sigma-Aldrich, St. Louis, MI, USA) supplemented with Pierce™ Universal Nuclease (Thermo Fisher Scientific, Waltham, MA, USA). The homogenates were subsequently sonicated and centrifuged, and the resulting supernatants were collected. Protein concentration was determined using the bicinchoninic acid (BCA) assay, and all samples were normalized to a final concentration of 1 µg/mL.

Samples were then subjected to in-gel enzymatic digestion [39]. Briefly, proteins were immobilized within a polyacrylamide gel matrix, reduced with dithiothreitol, and alkylated with iodoacetamide. Proteolytic digestion was performed overnight using trypsin (Pierce™ Trypsin Protease, Thermo Fisher Scientific, Waltham, MA, USA). The resulting peptides were extracted from the gel and purified using C18 ZipTip columns (Merck Millipore, Darmstadt, Germany) according to the manufacturer’s instructions. Peptide concentration was measured using a NanoDrop spectrophotometer (Thermo Fisher Scientific, Waltham, MA, USA), and samples were normalized before UHPLC-HRMS injection.

UHPLC-HRMS analysis was performed as previously described [40]. Briefly, peptide samples were injected into an Easy-nLC 1200 UHPLC system (Thermo Fisher Scientific, Waltham, MA, USA) coupled to a Q Exactive™ HF-X Hybrid Quadrupole-Orbitrap mass spectrometer (Thermo Fisher Scientific, Waltham, MA, USA). Peptides were initially loaded onto a trap column and subsequently separated on a 50 cm analytical column using a 180 min linear gradient at a flow rate of 300 nL min^−1^. Mass spectrometric data were acquired in positive electrospray ionization mode using a data-dependent acquisition (DDA) method to obtain MS/MS spectra.

The acquired raw data were processed and analyzed using Proteome Discoverer™ version 2.5 (Thermo Fisher Scientific, Waltham, MA, USA). MS/MS spectra were searched using the Sequest HT^®^ search engine against the Mus musculus Swiss-Prot canonical database (UniProt, version 2025-06-18; 17,225 sequences). Protein identifications were validated using the Percolator^®^ algorithm, applying a strict false discovery rate (FDR) threshold of 1% at the protein level. Only high-confidence protein identifications were retained for downstream analysis, defined as master proteins within each protein group and supported by the identification of at least two unique peptides.

Label-free quantification was performed using the Minora Feature Detector implemented in Proteome Discoverer™ version 2.5, with protein abundances calculated from precursor ion intensities. Normalization across samples was initially carried out based on total peptide abundance. Subsequently, quantitative data were further normalized using a median-based approach applied to the log_2_ ratio values between experimental conditions.

Protein abundance ratios were calculated directly from aggregated protein abundances. Hypothesis testing was performed using a t-test based on the background population of identified proteins and peptides. Proteins were considered differentially expressed when *p* < 0.01. To classify proteins as over- or under-expressed following treatment, log_2_-transformed abundance ratios were used, with values > 1 indicating upregulation and values < −1 indicating downregulation.

Functional enrichment analysis of significantly deregulated proteins was performed using GeneCodis, a web-based tool that integrates multiple annotation sources to identify significantly enriched Gene Ontology (GO) terms, functional categories, and biological pathways within gene or protein lists [41]. GeneCodis enables the simultaneous analysis of single and combined annotations, providing a comprehensive overview of the biological processes associated with the differentially expressed proteins (https://genecodis.genyo.es, accessed on 22 October 2025). Visualization of overlaps among differentially expressed protein sets was performed using the Intervene web application, which generates UpSet plots to represent shared and condition-specific protein signatures [42]. Functional pathway enrichment was further evaluated using the Kyoto Encyclopedia of Genes and Genomes (KEGG) database (http://www.genome.jp/kegg/pathway.html, accessed on 22 October 2025).

### 4.7. mRNA Isolation and qRT-PCR Analysis

Total mRNA was isolated from liver tissue samples (30–50 mg) using Trizol^®^ reagent (Invitrogen, Carlsbad, CA, USA, #15596026) following the manufacturer’s protocol. The concentration and purity of the extracted mRNA were measured with a Nanodrop™ ND-1000 spectrophotometer (Thermo Fisher Scientific, Waltham, MA, USA), ensuring A260/A280 ratios between 1.8 and 2.0. Subsequently, 1 μg of mRNA was reverse transcribed into cDNA using the Transcriptor Reverse Transcriptase kit with random hexamer primers (Transcriptor Universal cDNA Master, Roche Diagnostic, Mannheim, Germany, #5893151001). Amplification of cDNA was performed in a final reaction volume of 10 µL, consisting of 4.5 µL of cDNA (pre-diluted 1:100) and 5.5 µL of reaction mixture (PerfeCTa qPCR ToughMix, Quantabio, Beverly, CA, USA, #733-2091), which included the corresponding rat-specific probe (TaqMan^®^ Gene Expression Assays, Thermo Fisher Scientific, Waltham, MA, USA). Quantitative real-time polymerase chain reaction (qRT-PCR) assays and relative gene expression analysis were carried out using a CFX Duet Real-Time PCR Detection System (Bio-Rad, Hercules, CA, USA) as previously described [43]. Primer sequences for qRT-PCR were selected based on the Thermo Fisher Scientific TaqMan^®^ Gene Expression Assays database (https://www.thermofisher.com/taqman/gene-expression/assay/query) (accessed on 5 February 2026) (the Supplementary Table S4 shows primers for qPCR gene expression analysis). Gene expression levels were normalized using β-actin as the reference housekeeping gene.

### 4.8. In Vivo Evaluation of Wound-Healing Activity Using the Zebrafish (Danio rerio) Model

#### 4.8.1. Zebrafish Maintenance and Experimental Design

Adult zebrafish (*Danio rerio*) of age 7–8 months were used in this study. Fish were maintained under standard laboratory conditions (28 ± 1 °C, 14:10 h light/dark cycle) in a recirculating aquatic system and fed twice daily. All experimental procedures were conducted in accordance with institutional and international guidelines for animal care and use and the ARRIVE guidelines [38]. Fish were randomly divided into three experimental groups: Calvatin 2% (cream base containing 2% PsBu), PBS (physiological control), and cream base alone (CB), with nine fish per group (*n* = 9). This design allowed direct attribution of any observed effects to the polysaccharide component rather than to the vehicle formulation.

#### 4.8.2. Laser-Induced Wound Model

Cutaneous wounds were generated using a laser-induced injury model, a well-established method for studying tissue regeneration in zebrafish [19,44]. After anesthesia with tricaine methanesulfonate (MS-222), each fish received four laser pulses of 5 mJ applied to the black pigmented band of the trunk, ensuring reproducible wound size and depth. An Nd:YAG (Neodymium-doped Yttrium Aluminum Garnet) laser was used as the excitation source. This model allows precise induction of epithelial damage while preserving fish viability and is widely used to investigate wound-healing kinetics and regenerative responses in vivo [21,45,46].

#### 4.8.3. Treatment Protocol

Immediately after laser injury, treatments were applied topically to the wounded area. During application, fish were positioned so that their bodies remained exposed to air for 4 min, allowing optimal skin absorption of the test substances. To maintain adequate respiration and minimize stress, the head of each fish remained immersed in an anesthetic solution throughout the procedure.

Treatments were administered following a procedure by other authors [21], applied daily during the first two days post-laser injury, followed by a 4-day recovery period to reduce stress related to anesthesia and manipulation, then every two days, and subsequently every four days once wound healing was observed. Treatment was stopped once complete wound closure was achieved in most individuals, indicating full re-epithelialization.

#### 4.8.4. Image Acquisition and Wound-Healing Analysis

Wound images were captured at predefined time points using the ToupView imaging system under standardized lighting and magnification conditions of a Nikon Microphot-FX Fluorescence Microscope (Nikon DS-L1 camera) (Nikon Europe B.V., Amstelveen, The Netherlands). Quantitative analysis of wound area was performed using ImageJ software (version 1.54g). Wound-closure percentage was calculated by measuring the reduction in wound area relative to the initial post-injury area [21]. Healing kinetics of wound closure were measured as the percentage of wound area of each specimen at 1, 4, 6, 8, 14, 18, 21, 25, 28, and 32 days post-laser irradiation. Area percentage was preferred to total area (mm^2^) to eliminate the slight differences in wound areas observed on the first days post-irradiation.

### 4.9. Statistical Analysis

To assess differences in healing rate and overall regenerative efficiency, the mean and standard deviation of wound areas were calculated for each treatment group. Normality of data distribution was assessed using the Shapiro–Wilk test (Statgraphics Centurion 19). Pairwise comparisons between treatment groups were performed using the one-way ANOVA test and the post hoc Fisher’s LSD test (Statgraphics Centurion 19).

## 5. Conclusions

This study demonstrates that polysaccharides extracted from *Bovistella utriformis*, formulated as Calvatin 2%, exert a significant pro-healing effect across complementary in vivo models. In the murine excisional wound model, topical treatment accelerated wound contraction and re-epithelialization compared with both saline and vehicle-treated groups. These macroscopic improvements were accompanied by treatment-associated proteomic changes, which revealed a treatment-specific remodeling of the wound microenvironment. Calvatin induced the upregulation of proteins associated with the innate immune response, complement and coagulation cascades, and acute-phase processes, while downregulating proteins mainly related to lipid metabolism, mitochondrial energy production, and contractile functions. This coordinated profile suggests the involvement of immune- and hemostasis-related pathways together with metabolic reprogramming during early-to-intermediate stages of repair. Importantly, the limited proteomic impact of the base cream confirms that the observed biological effects are primarily attributable to the fungal polysaccharide fraction. The zebrafish laser-induced wound model further validated these findings, showing earlier onset and faster progression of wound closure under Calvatin treatment compared with controls. Moreover, chronic oral administration of PsBu did not induce major hepatic inflammatory alterations, supporting a favorable safety profile under the tested conditions. Overall, the integration of macroscopic, proteomic, and regenerative data indicates that *B. utriformis* polysaccharides are associated with a biologically regulated wound-healing response involving immune-related proteomic changes. These findings support their potential development as mechanistically grounded natural agents for topical regenerative therapies.

## Data Availability

The original contributions presented in this study are included in the article/Supplementary Materials. Further inquiries can be directed to the corresponding author(s).

## Supplementary Materials

The following supporting information can be downloaded at: https://www.mdpi.com/article/10.3390/molecules31081233/s1. Supplementary Figure S1 shows the KEGG pathway map of Complement and Coagulation Cascades (hsa04610), highlighting deregulated proteins in wound tissue following Calvatin treatment. Supplementary Figure S2 presents the KEGG pathway map of Neutrophil Extracellular Trap formation (hsa04613). Supplementary Table S1 provides the complete list of differentially expressed proteins. Supplementary Table S2 lists the 41 deregulated proteins mapped to the Complement and Coagulation Cascades pathway. Supplementary Table S3 contains the deregulated proteins mapped to the NET Formation pathway. Supplementary Table S4 presents the primer references for TaqMan^®^ Gene Expression Assays. Supplementary Materials S1 contain supplementary data related to the phylogenetic analysis. Supplementary Materials S2 include supplementary data related to the cream base formulation.

## Abbreviations

The following abbreviations are used in this manuscript:

Abbreviation	Full term
ARRIVE	Animal Research: Reporting of In Vivo Experiments
ATB	Antibiotic
BCA	Bicinchoninic Acid
PsBu	Polysaccharides from Bovistella utriformis
CB	Cream Base
cDNA	Complementary DNA
DDA	Data-Dependent Acquisition
DNA	Deoxyribonucleic Acid
FDR	False Discovery Rate
GO	Gene Ontology
HRMS	High-Resolution Mass Spectrometry
Ikbkb	Inhibitor of Nuclear Factor Kappa B Kinase Subunit Beta
IL	Interleukin
IL-6, IL-1β	Interleukin 6, Interleukin 1 beta
*Il-6*, *Il-1β*	Genes encoding Interleukin 6 and 1 beta (genes)
Igf1	Insulin-Like Growth Factor 1
INCI	International Nomenclature of Cosmetic Ingredients
ip	Intraperitoneal
ITS	Internal Transcribed Spacer
KEGG	Kyoto Encyclopedia of Genes and Genomes
MAPK	Mitogen-Activated Protein Kinase
mRNA	Messenger Ribonucleic Acid
MS-222	Tricaine Methanesulfonate
NET	Neutrophil Extracellular Trap
NF-κB	Nuclear Factor Kappa B
PBS	Phosphate-Buffered Saline
PCR	Polymerase Chain Reaction
PI3K/Akt	Phosphoinositide 3-Kinase / Protein Kinase B
Pparγ	Peroxisome Proliferator-Activated Receptor Gamma
PVPP	Polyvinylpolypyrrolidone
qRT-PCR	Quantitative Real-Time Polymerase Chain Reaction
RIPA	Radioimmunoprecipitation Assay Buffer
SEM	Standard Error of the Mean
Spsb3	SplA/Ryanodine Receptor Domain and SOCS Box Containing 3
Stat3	Signal Transducer and Activator of Transcription 3
TNF-α	Tumor Necrosis Factor Alpha
*Tnf-α*	gene encoding Tumor Necrosis Factor Alpha (gen)
UHPLC	Ultra-High-Performance Liquid Chromatography
