# Supplementary material for: Integrative In Vivo and Proteomic Analysis of a Bovistella utriformis Polysaccharide Formulation Reveals Mechanisms of Enhanced Skin Wound Healing
Source: Molecules. 2026 Apr 8;31(8):1233. doi: 10.3390/molecules31081233 (PMC13119201; doi:10.3390/molecules31081233)
Supplement: Supplementary file 1 [file molecules-31-01233-s001.zip › Supplementary Table S2.pdf]

Table S2. List of 41 differentially deregulated proteins mapped to the KEGG “Complement and Coagulation Cascades” pathway.

| **Gene Symbol** | **Protein Full Name** |
|---|---|
| Fgb | Fibrinogen beta chain |
| C8b | Complement component 8, beta polypeptide |
| Serpinc1 | Antithrombin (serpin clade C member 1) |
| C1qa | Complement C1q subcomponent alpha chain |
| C1qb | Complement C1q subcomponent beta chain |
| C1qc | Complement C1q subcomponent C chain |
| C2 | Complement C2 |
| C3 | Complement C3 |
| C4b | Complement C4B |
| C4bp | Complement component 4 binding protein |
| C5ar1 | Complement component 5a receptor 1 |
| C6 | Complement C6 |
| C9 | Complement C9 |
| Cfh | Complement factor H |
| Cfi | Complement factor I |
| Clu | Clusterin |
| F10 | Coagulation factor X |
| F13b | Coagulation factor XIII beta subunit |
| F5 | Coagulation factor V |
| Fga | Fibrinogen alpha chain |
| Hc | Hemolytic complement |
| Itgb2 | Integrin beta 2 |
| Itgb2l | Integrin beta 2-like |
| Klkb1 | Plasma kallikrein |
| Kng1 | Kininogen 1 |
| Masp1 | MBL-associated serine protease 1 |
| Masp2 | MBL-associated serine protease 2 |
| Mbl2 | Mannose-binding lectin 2 |
| Serpinb2 | Serpin clade B member 2 |
| Plg | Plasminogen |
| Proc | Protein C |
| Pros1 | Protein S (alpha) |
| Serpina1d | Serpin clade A member 1D |
| Vtn | Vitronectin |
| Vwf | Von Willebrand factor |
| C8a | Complement component 8 alpha chain |
| C1s1 | Complement C1s subcomponent |

| Gene Symbol | Protein Full Name |
|---|---|
| Cpb2 | Carboxypeptidase B2 (TAFI) |
| F12 | Coagulation factor XII (Hageman factor) |
| C8g | Complement component 8 gamma chain |
| Fgg | Fibrinogen gamma chain |
