# Supplementary material for: Integrative In Vivo and Proteomic Analysis of a Bovistella utriformis Polysaccharide Formulation Reveals Mechanisms of Enhanced Skin Wound Healing
Source: Molecules. 2026 Apr 8;31(8):1233. doi: 10.3390/molecules31081233 (PMC13119201; doi:10.3390/molecules31081233)
Supplement: Supplementary file 1 [file molecules-31-01233-s001.zip › Supplementary Table S3.pdf]

Supplementary Table S3. Differentially deregulated proteins mapped to the KEGG "Neutrophil extracellular trap formation" pathway.

Gene Symbol	Protein Full Name
H4c17	H4 clustered histone 17
Fgb	Fibrinogen beta chain
C3	Complement component 3
C5ar1	Complement component 5a receptor 1
Camp	Cathelicidin antimicrobial peptide
Ctsg	Cathepsin G
Cyba	Cytochrome b-245 alpha chain
Cybb	Cytochrome b-245 beta chain
Fga	Fibrinogen alpha chain
Fpr2	Formyl peptide receptor 2
Hc	Hemolytic complement
Itgal	Integrin alpha L
Itgb2	Integrin beta 2
Itgb2l	Integrin beta 2-like
Mpo	Myeloperoxidase
Ncf1	Neutrophil cytosolic factor 1
Ncf2	Neutrophil cytosolic factor 2
Ncf4	Neutrophil cytosolic factor 4
Padi4	Peptidyl arginine deiminase type IV
Rac2	Rac family small GTPase 2
Vwf	Von Willebrand factor
H4c3	H4 clustered histone 3
H4c4	H4 clustered histone 4
H4c6	H4 clustered histone 6
H4c9	H4 clustered histone 9
H4c11	H4 clustered histone 11
H4c12	H4 clustered histone 12
H4c18	H4 clustered histone 18
H2ac25	H2A clustered histone 25
H2bc3	H2B clustered histone 3
H4c16	H4 histone 16
H4c1	H4 clustered histone 1
H4c2	H4 clustered histone 2
Macroh2a2	MacroH2A.2 histone variant
Elane	Neutrophil elastase
H4c8	H4 clustered histone 8
Slc25a31	Solute carrier family 25 member 31 (adenine nucleotide translocator 4)
H2az2	H2A.Z histone variant 2

Gene Symbol	Protein Full Name
H4c14	H4 clustered histone 14
Fgg	Fibrinogen gamma chain
