# Supplementary material for: Integrative In Vivo and Proteomic Analysis of a Bovistella utriformis Polysaccharide Formulation Reveals Mechanisms of Enhanced Skin Wound Healing
Source: Molecules. 2026 Apr 8;31(8):1233. doi: 10.3390/molecules31081233 (PMC13119201; doi:10.3390/molecules31081233)
Supplement: Supplementary file 1 [file molecules-31-01233-s001.zip › Supplementary Table S4.pdf]

Supplementary Table 4. Primer references for TaqMan® Gene Expression Assays (Applied Biosystems, Waltham, USA).

Gene Symbol	Assay ID	GenBank Accession Number	Amplicon Length (bp)
Gapdh	Mm99999915_g1	NM_001289726.1	107
Tnf-α	Mm00443258_m1	NM_001278601.1	81
Il-6	Mm00446190_m1	NM_031168.1	78
Il-1β	Mm99999061_mH	NM_008361.3	99
Ikbkb	Mm01222247_m1	NM_001159774.1	63
Nkiras	Mm01310378_m1	NM_023526.3	95
Spsb3	Mm00466126_m1	NM_001163750.1	77
Pparγ	Mm00440940_m1	NM_011146.3	63
Stat3	Mm01219775_m1	NM_011486.4	75
Igf1	Mm00439560_m1	NM_001111274.1	77
Gpx1	Mm04207457_g1	NM_008160.6	78
Gss	Mm00515065_m1	NM_001291111.1	67
Gclc	Mm00802658_m1	NM_010295.2	78

Abbreviations: *Gapdh*: Glyceraldehyde-3-phosphate dehydrogenase, *Tnf-α*: tumor necrosis factor alpha, *Il-6*: Interleukin 6, *Il-1β*: Interleukin 1 beta, *Ikbkb*: inhibitor of nuclear factor kappa B kinase subunit beta, *Nkiras*: NFKB inhibitor interacting Ras-like protein 1, *Spsb3*: splA/ryanodine receptor domain and SOCS box containing 3, *Pparγ*: Peroxisome proliferator activated receptor gamma, *Stat3*: Signal transducer and activator of transcription 3, *Igf1*: insulin-like growth factor 1, *Gpx1*: glutathione peroxidase 1, *Gss*: glutathione synthetase and, *Gclc*: Glutamate-cysteine ligase, catalytic subunit.
