# Supplementary material for: Integrative In Vivo and Proteomic Analysis of a Bovistella utriformis Polysaccharide Formulation Reveals Mechanisms of Enhanced Skin Wound Healing
Source: Molecules. 2026 Apr 8;31(8):1233. doi: 10.3390/molecules31081233 (PMC13119201; doi:10.3390/molecules31081233)
Supplement: Supplementary file 1 [file molecules-31-01233-s001.zip › supplementary material S2 de la crema base/ecologic certificate.pdf]

# FICHA DE DADOS DE SEGURANÇA
(de acordo com o Regulamento (UE) 2020/878)

# PB01-Crema Base Certificada ecológica



## SECÇÃO 1: IDENTIFICAÇÃO DA SUBSTÂNCIA/MISTURA E DA SOCIEDADE/EMPRESA.

**1.1 Identificador do produto.**

Nome do produto: Crema Base Certificada ecológica
Código do produto: PB01

**1.2 Utilizações identificadas relevantes da substância ou mistura e utilizações desaconselhadas.**

Materia prima para cosmética.

**Usos não aconselhados:**
Usos diferentes aos aconselhados.

**1.3 Identificação do fornecedor da ficha de dados de segurança.**

Empresa: **Camassia ECO s.l.**
Endereço: Calle V, Nave 26, Pol.Ind.La Redonda
População: 04710 - Santa María del Aguila
Distrito: Almeria
Telefone: 950 57 61 62
E-mail: info@cremas-caseras.es
Web: www.cremas-caseras.es

**1.4 Número de telefone de emergência:** Instituto Nacional De Toxicología y Ciencias Forenses 915620420 (Disponível 24h)
Em caso de intoxicação contactar o Centro de Informação Antivenenos (CIAV) (+351) 800 250 250.
Atendimento médico 24 horas por dia, 7 dias por semana.

## SECÇÃO 2: IDENTIFICAÇÃO DOS PERIGOS.

**2.1 Classificação da substância ou mistura.**
O produto não é classificado como perigoso segundo o Regulamento (CE) No 1272/2008.

**2.2 Elementos do rótulo.**
Este producto no esta clasificado como peligroso conforme a la aplicación del Reglamento 1272/2008 y sus actualizaciones.

**2.3 Outros perigos.**

A mistura não contém substâncias classificadas como PBT.
A mistura não contém substâncias classificadas como mPmB.
A mistura não contém substâncias com propriedades desreguladoras do sistema endócrino.

Em condições de uso normal e na sua forma original, o produto não tem efeitos negativos sobre a saúde e o meio ambiente.

## SECÇÃO 3: COMPOSIÇÃO/INFORMAÇÃO SOBRE OS COMPONENTES.

**3.1 Substâncias.**
Não Aplicável.

**3.2 Misturas.**

*-Continua na página seguinte.-*

Substâncias que representam um perigo para a saúde ou o meio ambiente de acordo com a Regulamento (CE) No. 1272/2008, têm atribuído um limite de exposição comunitário no lugar de trabalho, estão classificadas como PBT/ mPmB ou incluídas na Lista de Candidatos:

| Identificadores | Nome | Concentração | (*)Classificação -Regulamento 1272/2008 | |
|---|---|---|---|---|
| | | | Classificação | Limite de concentração específico e a Estimativa da Toxicidade Aguda |
| N. CAS: 56-81-5<br>N. CE: 200-289-5<br>N. registo: 01-2119471987-18-XXXX | [2] glycerol | 2.5 - 4.99 % | - | - |

(*)*O texto completo das frases H é pormenorizado na secção 16 desta Ficha de Segurança.*
*[2] Substância com limite nacional de exposição no local de trabalho (ver secção 8.1).*

## SECÇÃO 4: MEDIDAS DE PRIMEIROS SOCORROS.

**4.1 Descrição das medidas de emergência.**
Devido à composição e à tipologia das substâncias presentes no produto, não são necessárias advertências específicas.

**Inalação.**
Em caso de paragem respiratória, procurar assistência médica urgente. Retirar o acidentado para o ar livre, mantê-lo em repouso, se a respiração for irregular ou se detiver, praticar respiração artificial.

**Contacto com os olhos.**
Retirar as lentes de contacto, se existirem e for fácil de o fazer. Lavar os olhos com água limpa e fresca e procurar ajuda médica.

**Contacto com a pele.**
Tirar a roupa contaminada.

**Ingestão.**
Mantê-lo em repouso. NUNCA provocar o vômito.

**4.2 Sintomas e efeitos mais importantes, tanto agudos como retardados.**
Não são conhecidos efeitos agudos e tardios da exposição ao produto.

**4.3 Indicações sobre cuidados médicos urgentes e tratamentos especiais necessários.**
Em caso de dúvida, ou quando persistirem os sintomas de mal-estar, solicitar ajuda médica. Não administrar nunca nada por via oral a pessoas que se encontrem inconscientes.

## SECÇÃO 5: MEDIDAS DE COMBATE A INCÊNDIOS.

**5.1 Meios de extinção.**
**Meios de extinção adequados:**
Pó extintor ou $CO_2$. Em caso de incêndios mais graves também espuma resistente ao álcool e água pulverizada.

**Meios de extinção inadequados:**
Não usar para a extinção jato direto de água. Em presença de tensão elétrica não é aceitável utilizar água ou espuma como meio de extinção.

*-Continua na página seguinte.-*

**5.2 Perigos especiais decorrentes da substância ou mistura.**
**Riscos especiais.**
A exposição aos produtos de combustão ou decomposição pode ser prejudicial para a saúde.

**5.3 Recomendações para o pessoal de combate a incêndios.**
Refrigerar com água os tanques, cisternas ou recipientes próximos à fonte de calor ou fogo. Ter em conta a direção do vento.

**Equipamento de proteção contra incêndios.**
Segundo a magnitude do incêndio, pode ser necessário o uso de roupas de proteção contra o calor, equipamento respiratório autónomo, luvas, óculos protetores ou máscaras faciais e botas.

## SECÇÃO 6: MEDIDAS EM CASO DE FUGA ACIDENTAL.

**6.1 Precauções individuais, equipamento de proteção e procedimentos de emergência.**
Para controlo de exposição e medidas de proteção individual, ver secção 8.

**6.2 Precauções a nível ambiental.**
Produto não classificado como perigoso para o meio ambiente; na medida do possível, evite qualquer derrame.

**6.3 Métodos e materiais de confinamento e limpeza.**
Conter e recolher o derrame com material absorvente inerte (terra, areia, vermiculita, terra de diatomáceas...) e limpe a área imediatamente com um descontaminante adequado.
Deposite os resíduos em recipientes fechados e adequados para a eliminação, de acordo com os regulamentos locais e nacionais (ver secção 13).

**6.4 Remissão para outras secções.**
Para controlo de exposição e medidas de proteção individual, ver secção 8.
Para a posterior eliminação dos resíduos, seguir as recomendações da secção 13.

## SECÇÃO 7: MANUSEAMENTO E ARMAZENAGEM.

**7.1 Precauções para um manuseamento seguro.**
O produto não exige medidas de manuseamento especiais; recomendam-se as seguintes medidas gerais:
Para a proteção pessoal, ver secção 8.
Na zona de trabalho deve ser proibido fumar, comer e beber.
Cumprir com a legislação sobre segurança e higiene no trabalho.
Não utilizar nunca pressão para esvaziar os recipientes, não são recipientes resistentes à pressão. Conservar o produto em recipientes de um material idêntico ao original.

**7.2 Condições de armazenagem segura, incluindo eventuais incompatibilidades.**
O produto não exige medidas especiais de armazenamento.
Como condições gerais de armazenamento, devem-se evitar fontes de calor, radiações, eletricidade e o contacto com alimentos.
Manter longe de agentes oxidantes e de materiais fortemente ácidos ou alcalinos.
Armazenar os recipientes entre 5 e 35 ºC, num local seco e bem ventilado.
Armazenar segundo a legislação local. Observar as indicações do rótulo. Depois de ter aberto os recipientes, estes devem ser fechados de novo com cuidado, e colocados verticalmente para evitar derrames.

O produto não está afetado pela Directiva 2012/18/UE (SEVESO III).

**7.3 Utilizações finais específicas.**

*-Continua na página seguinte.-*

No especificado.

## SECÇÃO 8: CONTROLO DA EXPOSIÇÃO/PROTEÇÃO INDIVIDUAL.

### 8.1 Parâmetros de controlo.

Limite de exposição durante o trabalho para:

| Nome | N. CAS | País | Valor-limite | ppm | mg/m³ |
|---|---|---|---|---|---|
| glycerol | 56-81-5 | Portugal [1] | **Oito horas** | 2,7 | 10 |
| | | | **Curta duração** | | |

*[1] De acordo com a Norma Portuguesa 1796 adotou pelo Instituto português de qualidade.*

O produto NÃO contém substâncias com Valores Biológicos Limite.
Níveis de concentração DNEL/DMEL:

| Nome | DNEL/DMEL | Tipo | Valor |
|---|---|---|---|
| glycerol<br>N. CAS: 56-81-5<br>N. CE: 200-289-5 | DNEL<br>(Trabalhadores) | Inalação, Crónico, Efeitos locais | 56<br>(mg/m³) |

DNEL: Derived No Effect Level, (nível sem efeito obtido) nível de exposição à substância por baixo do qual não são previstos efeitos adversos.
DMEL: Derived Minimal Effect Level, nível de exposição que corresponde a um risco baixo, que deve ser considerado um risco mínimo tolerável.

### 8.2 Controlo da exposição.

**Medidas de ordem técnica:**

| **Concentração:** | **100 %** |
|---|---|
| **Usos:** | **Materia prima para cosmética.** |
| **Proteção respiratória:** | |
| Se as medidas técnicas recomendadas forem cumpridas, não é necessário qualquer equipamento de proteção individual. | |
| **Proteção das mãos:** | |
| Se o produto for manuseado corretamente, não é necessário qualquer equipamento de proteção individual. | |
| **Proteção dos olhos:** | |
| Se o produto for manuseado corretamente, não é necessário qualquer equipamento de proteção individual. | |
| **Proteção da pele:** | |
| EPI: | Calçado de trabalho |
| Características: | Marcação «CE» Categoria II. |
| Normas CEN: | EN ISO 13287, EN 20347 |
| Manutenção: | Estes artigos adaptam-se à forma do pé do primeiro utilizador. Por este motivo, e igualmente por questões de higiene, deve-se evitar a sua reutilização por qualquer outra pessoa. |
| Observações: | O calçado de trabalho para uso profissional é o que incorpora elementos de protecção destinados à protecção do utilizador contra as lesões que possam provocar acidentes |

*-Continua na página seguinte.-*

## SECÇÃO 9: PROPRIEDADES FÍSICO-QUÍMICAS.

**9.1 Informações sobre propriedades físicas e químicas de base.**
Estado físico: Sólido
Cor: blanco
Odor: característico
Limiar de odor: Não aplicável/Não disponível devido à natureza/propriedades do produto
Ponto de fusão: Não aplicável/Não disponível devido à natureza/propriedades do produto
Ponto de congelação: Não aplicável/Não disponível devido à natureza/propriedades do produto
Ponto de ebulição ou ponto de ebulição inicial e intervalo de ebulição: Não aplicável/Não disponível devido à natureza/propriedades do produto
Inflamabilidade: Não aplicável/Não disponível devido à natureza/propriedades do produto
Limite inferior de explosividade: Não aplicável/Não disponível devido à natureza/propriedades do produto
Limite superior de explosividade: Não aplicável/Não disponível devido à natureza/propriedades do produto
Ponto de inflamação: 248 ºC
Temperatura de autoignição: Não aplicável/Não disponível devido à natureza/propriedades do produto
Temperatura de decomposição: Não aplicável/Não disponível devido à natureza/propriedades do produto
pH: 5.5 (10%)
Viscosidade cinemática: Não aplicável/Não disponível devido à natureza/propriedades do produto
Solubilidade: Não aplicável/Não disponível devido à natureza/propriedades do produto
Hidrosolubilidade: Não aplicável/Não disponível devido à natureza/propriedades do produto
Liposolubilidade: Não aplicável/Não disponível devido à natureza/propriedades do produto
Coeficiente de partição n-octanol/água (valor logarítmico): Não aplicável/Não disponível devido à natureza/propriedades do produto
Pressão de vapor: Não aplicável/Não disponível devido à natureza/propriedades do produto
Densidade absoluta: Não aplicável/Não disponível devido à natureza/propriedades do produto
Densidade relativa: 0.950 - 1.050
Densidade relativa do vapor: Não aplicável/Não disponível devido à natureza/propriedades do produto
Características das partículas: Não aplicável/Não disponível devido à natureza/propriedades do produto

**9.2 Outras informações.**
Não aplicável/Não disponível devido à natureza/propriedades do produto

## SECÇÃO 10: ESTABILIDADE E REATIVIDADE.

**10.1 Reatividade.**
O produto não apresentar riscos devido à sua reactividade.

**10.2 Estabilidade química.**
Estável sob as condições de manipulação e armazenamento recomendadas (ver epígrafe 7).

**10.3 Possibilidade de reações perigosas.**
O produto não tem a possibilidade de reacções perigosas.

**10.4 Condições a evitar.**
Evitar qualquer tipo de manipulação incorreta.

**10.5 Materiais incompatíveis.**
Manter afastado de agentes oxidantes e de materiais fortemente alcalinos ou ácidos, com o fim de evitar reacções exotérmicas.

**10.6 Produtos de decomposição perigosos.**
Não se decompõe se for destinado aos usos previstos.

*-Continua na página seguinte.-*

## SECÇÃO 11: INFORMAÇÃO TOXICOLÓGICA.

**11.1 Informações sobre as classes de perigo, tal como definidas no Regulamento (CE) nº 1272/2008.**
Não existem dados disponíveis ensaiados do produto.
a) Toxicidade aguda;
Dados não conclusivos para a classificação.

b) Corrosão/irritação cutânea;
Dados não conclusivos para a classificação.

c) Lesões oculares graves/irritação ocular;
Dados não conclusivos para a classificação.

d) Sensibilização respiratória ou cutânea;
Dados não conclusivos para a classificação.

e) Mutagenicidade em células germinativas;
Dados não conclusivos para a classificação.

f) Carcinogenicidade;
Dados não conclusivos para a classificação.

g) Toxicidade reprodutiva;
Dados não conclusivos para a classificação.

h) Toxicidade para órgãos-alvo específicos (STOT) - exposição única;
Dados não conclusivos para a classificação.

i) Toxicidade para órgãos-alvo específicos (STOT) - exposição repetida;
Dados não conclusivos para a classificação.

j) Perigo de aspiração.
Dados não conclusivos para a classificação.

**11.2 Informações sobre outros perigos.**
**Propriedades desreguladoras do sistema endócrino**
Este produto não contém componentes com propriedades desreguladoras do sistema endócrino com efeitos sobre a saúde humana.
**Outras informações**
Não existem informações disponíveis sobre outros efeitos adversos para a saúde.

## SECÇÃO 12: INFORMAÇÃO ECOLÓGICA.

**12.1 Toxicidade.**
Não estão disponíveis informações relativas à Ecotoxicidade das substâncias presentes.

**12.2 Persistência e degradabilidade.**
Não se dispõe de informação relativa à biodegradabilidade das substâncias presentes.
Não se dispõe de informação relativa à degradabilidade das substâncias presentes.
Não há informação disponível sobre a persistência e degradabilidade do produto

*-Continua na página seguinte.-*

**12.3 Potencial de bioacumulação.**
**Informações relativas à Bioacumulação das substâncias presentes.**

| Nome | | Bioacumulação | | | |
|---|---|---|---|---|---|
| | | Log Pow | BCF | NOECs | Nível |
| glycerol<br>N. CAS: 56-81-5 | N. CE: 200-289-5 | -1,76 | - | - | Muito baixo |

**12.4 Mobilidade no solo.**
Não há informação disponível sobre a mobilidade no solo.
Não é permitida a descarga nos esgotos ou cursos de agua.
Evitar a penetração no solo.

**12.5 Resultados da avaliação PBT e mPmB.**
Não há informações disponíveis sobre a avaliação PBT e mPmB do produto.

**12.6 Propriedades desreguladoras do sistema endócrino.**
Este produto não contém componentes com propriedades desreguladoras do sistema endócrino sobre o ambiente.

**12.7 Outros efeitos adversos.**
O produto não é afetado pelo Regulamento (CE) nº 1005/2009 do Parlamento Europeu e do Conselho, de 16 de Setembro de 2009, relativo às substâncias que empobrecem a camada de ozono.
Não há informação sobre outros efeitos adversos para o meio ambiente.

## SECÇÃO 13: CONSIDERAÇÕES RELATIVAS À ELIMINAÇÃO.

**13.1 Métodos de tratamento de resíduos.**
Não é permitida a descarga em sumidouros ou cursos de água Os resíduos e recipientes vazios devem ser manipulados e eliminados de acordo com as legislações locais/nacionais vigentes.
Siga as disposições da Directiva (UE) 2018/851 relativa aos resíduos, Decreto-Lei n.º 102-D/2020 e Decisão da Comissão 2014/955 / UE (códigos LER), nas suas redações atuais.

## SECÇÃO 14: INFORMAÇÕES RELATIVAS AO TRANSPORTE.

Não é perigoso no transporte. Em caso de acidente e derrame do produto, actuar de acordo com o ponto 6.

*-Continua na página seguinte.-*

**14.1 Número ONU ou número de ID.**
Não é perigoso no transporte.

**14.2 Designação oficial de transporte da ONU.**
Descrição:
ADR/RID: Não é perigoso no transporte.
IMDG: Não é perigoso no transporte.
OACI/IATA: Não é perigoso no transporte.

**14.3 Classes de perigo para efeitos de transporte.**
Não é perigoso no transporte.

**14.4 Grupo de embalagem.**
Não é perigoso no transporte.

**14.5 Perigos para o ambiente.**
Não é perigoso no transporte.
Transporte por barco, FEm - Fichas de emergência (F – Incêndio, S - Derrames): Não Aplicável.

**14.6 Precauções especiais para o utilizador.**
Não é perigoso no transporte.

**14.7 Transporte marítimo a granel em conformidade com os instrumentos da OMI.**
Não é perigoso no transporte.

## SECÇÃO 15: INFORMAÇÃO SOBRE REGULAMENTAÇÃO.

**15.1 Regulamentação/legislação específica para a substância ou mistura em matéria de saúde, segurança e ambiente.**

Composto orgânico volátil (COV)
Teor de COV (p/p): 0 %
Teor de COV: 0 g/l

O produto não está afetado pelo Reglamento (UE) No 528/2012 relativo à comercialização e ao uso dos biocidas.
O produto não está afetado pelo procedimento estabelecido no Regulamento (UE) No 649/2012, relativo à exportação e importação de produtos químicos perigosos.

**15.2 Avaliação da segurança química.**
Não foi realizado uma avaliação da segurança química do produto.

## SECÇÃO 16: OUTRAS INFORMAÇÕES.

Modificações em relação à versão anterior:

- Modificações nos primeiros socorros (SECÇÃO 4.1).
- Modificações nas precauções de manuseamento e armazenagem (SECÇÃO 7.1).
- Modificação nos valores das propriedades físico-químicas (SECÇÃO 9).

*-Continua na página seguinte.-*

- Modificação da informação das condições de estabilidade e reatividade (SECÇÃO 10.2).
- Modificação da informação das condições de estabilidade e reatividade (SECÇÃO 10.3).
- Modificação da informação das condições de estabilidade e reatividade (SECÇÃO 10.4).
- Modificação da informação das condições de estabilidade e reatividade (SECÇÃO 10.5).
- Modificação da informação das condições de estabilidade e reatividade (SECÇÃO 10.6).

Classificação e procedimento utilizado para determinar a classificação das misturas em conformidade com o Regulamento (CE) n.º 1272/2008 [CRE]:

| | |
|---|---|
| Perigos físicos | Com base em dados de ensaio |
| Perigos para a saúde | Método de cálculo |
| Perigos para o ambiente | Método de cálculo |

Recomenda-se que só utilize o produto para os usos previstos.

**Informações sobre o Inventário TSCA (Toxic Substances Control Act) USA:**

| N. CAS | Nome | Estado |
|---|---|---|
| 56-81-5 | glycerol | Registrado |

Abreviaturas e siglas utilizadas:

| | |
|---|---|
| BCF: | Factor de bioconcentração. |
| CEN: | Comité Europeu de Normalização. |
| DMEL: | Derived Minimal Effect Level, nível de exposição que corresponde a um risco baixo, que deve ser considerado um risco mínimo tolerável. |
| DNEL: | Derived No Effect Level, (nível sem efeito obtido) nível de exposição à substância por baixo do qual não são previstos efeitos adversos. |
| EC50: | Concentração média eficaz. |
| EPI: | Equipamento de proteção individual. |
| LC50: | Concentração letal, 50%. |
| LD50: | Dose Letal, 50%. |
| NOEC: | Não se observou efeito de concentração. |

Principais referências bibliográficas e fontes de dados:

http://eur-lex.europa.eu/homepage.html
http://echa.europa.eu/
Regulamento (UE) 2020/878.
Regulamento (CE) No 1907/2006.
Regulamento (CE) No 1272/2008.


A informação facilitada nesta ficha de Dados de Segurança foi redigida de acordo com o REGULAMENTO (UE) 2020/878 DA COMISSÃO de 18 de junho de 2020 que altera o Anexo II do Regulamento (CE) n.o 1907/2006 do Parlamento Europeu e do Conselho relativo ao registo, avaliação, substâncias e misturas químicas (REACH).

A informação desta Ficha de Dados de Segurança do produto está baseada nos conhecimentos actuais e nas leis vigentes da CE e nacionais, quanto a que as condições de trabalho dos utilizadores estiverem fora do nosso conhecimento e controlo. O produto não deve ser utilizado para fins distintos àqueles que são especificados, sem ter primeiro uma instrução por escrito, da sua utilização. É sempre responsabilidade do utilizador tomar as medidas oportunas com a finalidade de cumprir com as exigências estabelecidas nas legislações.

*-Fim da ficha de dados de segurança.-*
