# Supplementary material for: Integrative In Vivo and Proteomic Analysis of a Bovistella utriformis Polysaccharide Formulation Reveals Mechanisms of Enhanced Skin Wound Healing
Source: Molecules. 2026 Apr 8;31(8):1233. doi: 10.3390/molecules31081233 (PMC13119201; doi:10.3390/molecules31081233)
Supplement: Supplementary file 1 [file molecules-31-01233-s001.zip › supplementary material S2 de la crema base/technical data sheet.pdf]

FICHA TÉCNICA

**NOMBRE DEL PRODUCTO:**	**Crema base**
**REFERENCIA DEL PRODUCTO:**	PB001
**INCI:**	aloe barbadensis leaf juice, caprylic triglyceride, glycerin, cetyl alcohol, cetearyl olivate, butyrospermum parkii butter, sorbitan olivate, glyceryl caprylate, tocopherol, xanthan gum, glyceryl undecilate, phytic acid, aqua benzyl alcohol
**CERTIFICACIÓN:**	COSMOS ORGANIC

**DESCRIPCIÓN**	
**Descripción:**	El 81% del total de los ingredientes procede de la agricultura ecológica<br>El 99% del total es de origen natural
**Nº CAS:**	85507-69-3/ 94349-62-9"/ 73398-61-5 // 65381-09-1 / 56-81-5 / 67762-27-0/ 85116-80-9/ 91697-49-3/ 348616-34-2/ 2778-96-3/ 194043-92-0 // 91080-23-8/ 223706-40-9/
**Nº EINECS:**	287-390-8/ 305-181-2/ 277-452-2// 265-724-3/ 200-289-5 / 267-008-6/ 293-515-7/ 247-668-1/ 234-394-2 / 236-935-8/ 201-506-6/ 231-791-2 / 202-859-9

**PARÁMETROS ORGANOLÉPTICOS**	
**Color:**	Blanco
**Apariencia:**	Crema

Made by dwit

CAMASSIA
PRODUCTOS PARA COSMÉTICA
Cosmética Natura Casera Shop
Nave 26, Pol.Ind La Redonda , El Ejido, Almeria 04710 Spain
Teléfono 0034 950 57 61 62
www.cremas-caseras.es

ESPECIFICACIONES TÉCNICAS

Densidad 0,956-1,056 g/ml
PH 5,3-5,6

ANÁLISIS MICROBIOLÓGICO

Total de bacterias: ≤ 500 UFC/g
Total de mohos y levaduras: ≤ 500 UFC/g
Sin patogenes

Made by dwit

CAMASSIA
PRODUCTOS PARA COSMÉTICA
Cosmética Natura Casera Shop
Nave 26, Pol.Ind La Redonda , El Ejido, Almeria 04710 Spain
Teléfono 0034 950 57 61 62
www.cremas-caseras.es

INFORMACIÓN ADICIONAL	
**Regulación EU de cosméticos:**	Permitido en productos cosméticos de acuerdo con lo establecido en el Reglamento de Cosméticos nº 231/2012 / CE y sus posterioresmodificaciones.
**Reach:**	N.D.
**Impurezas:**	N.D.
**Contaminantes:**	De acuerdo con todas las disposiciones legales aplicables en la UE para productos alimenticios y cosméticos.
**Solventes residuales:**	N.D.
**CMR:**	No contiene sustancias clasificadas como cancerígenas, mutagénicas o tóxicas para la reproducción (CMR) Según el Reglamento (CE)1272/2008.
**SVHC:**	Ninguna de las sustancias altamente preocupantes (SVHC)
**Testado en animales:**	Este producto no ha sido probado en experimentos con animales, no presenta riesgo de EEB / EET.
**BSE:**	No contiene materiales derivados de animales ni material relacionado con TSE (encefalopatías espongiformes transmisibles) y BSE(encefalopatía espongiforme bovina)
**GMO:**	Ausencia de organismos genéticamente modificados.
**Alérgenos:**	Sin alérgenos enumerados en el Anexo III de la Directiva de Cosméticos 76/768 / CEE, en sus posteriores modificaciones y en el nuevoReglamento de Cosméticos (CE) 1223/2009.
**Nanomateriales:**	Este producto no contiene nanopartículas ni sustancias tóxicas.
**Halal:**	N.D.
**Irradiación:**	Esto productos no ha sido sometido a radiación.
**Otros:**	N.D.
**Almacenamiento:**	De acuerdo con las disposiciones legales aplicables de la UE. Producto protegido de la luz, en recipientes completamente llenos y biencerrados.
**Envase:**	Tarro de HDPE blanco

Este documento, cualquier respuesta o información proporcionada aquí por Camassia ECO sl, no constituye una obligación legalmente vinculante de Camassia ECO sl. mientras que las descripciones, los diseños, los datos y la información contenidos en este documento se presentan de buena fe y se consideran precisos; se proporcionan únicamente para su orientación. Porque muchos factores pueden afectar el procesamiento o la aplicación / uso, le recomendamos que realice pruebas para determinar la idoneidad de un producto para su propósito particular antes de usar. No exime a nuestros clientes de la obligación de realizar una inspección completa de los productos en el momento de la entrega o de cualquier otra obligación

CAMASSIA
PRODUCTOS PARA COSMÉTICA

Cosmética Natura Casera Shop
Nave 26, Pol.Ind La Redonda , El Ejido, Almeria 04710 Spain
Teléfono 0034 950 57 61 62
www.cremas-caseras.es

Made by dwit
