# Supplementary material for: Integrative In Vivo and Proteomic Analysis of a Bovistella utriformis Polysaccharide Formulation Reveals Mechanisms of Enhanced Skin Wound Healing
Source: Molecules. 2026 Apr 8;31(8):1233. doi: 10.3390/molecules31081233 (PMC13119201; doi:10.3390/molecules31081233)
Supplement: Supplementary file 1 [file molecules-31-01233-s001.zip › Supplementary figures 1 and 2.pdf]

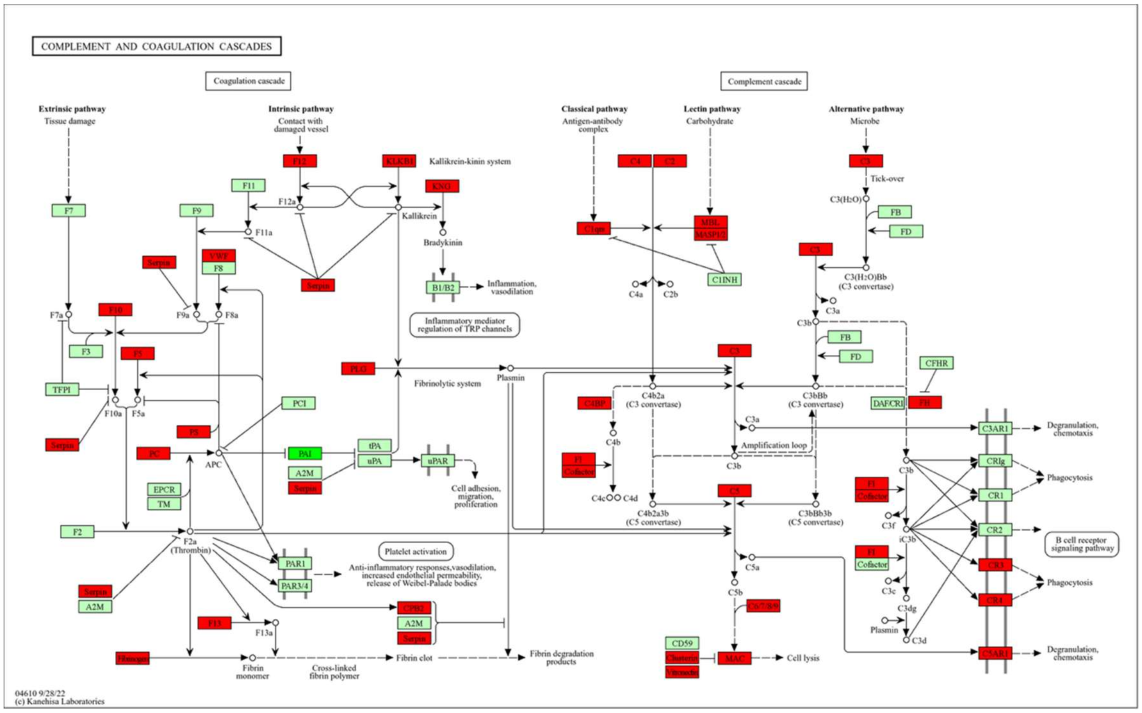

Supplementary Figure S1. KEGG pathway map of Complement and coagulation cascades highlighting deregulated proteins identified in wound tissue following treatment with B. utriformis–derived polysaccharides. Proteins labeled in red correspond to overexpressed proteins, whereas proteins labeled in dark green indicate underexpressed proteins based on the proteomic analysis. This pathway included 41 deregulated proteins, encompassing key components of the complement system, coagulation cascade, fibrinolysis, and regulatory mechanisms.

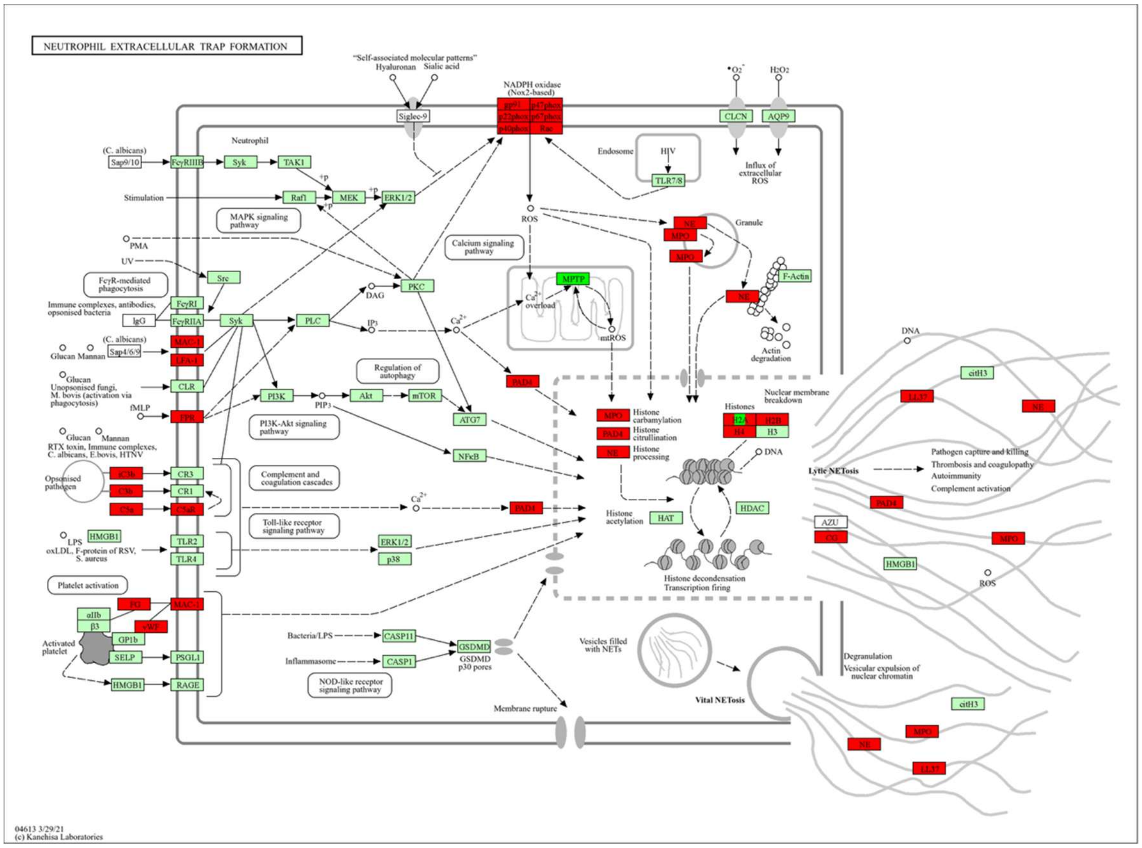

Supplementary Figure S2. KEGG pathway map of Neutrophil extracellular trap (NET) formation showing deregulated proteins identified in wound tissue following treatment with B. utriformis–derived polysaccharides. Proteins labeled in red indicate overexpressed proteins, while proteins labeled in dark green represent underexpressed proteins. Highlighted proteins correspond to components involved in neutrophil activation, reactive oxygen species production, chromatin remodeling, histone modification, and granule-derived effector functions. This pathway comprised 40 deregulated proteins identified in the proteomic analysis.
