# Supplementary material for: Integrative In Vivo and Proteomic Analysis of a Bovistella utriformis Polysaccharide Formulation Reveals Mechanisms of Enhanced Skin Wound Healing
Source: Molecules. 2026 Apr 8;31(8):1233. doi: 10.3390/molecules31081233 (PMC13119201; doi:10.3390/molecules31081233)
Supplement: Supplementary file 1 [file molecules-31-01233-s001.zip › Supplementary Table S1 (1).pdf]

Gene Symbol	Accession	Description	Abundance Ratio: (Calvatia cream) / (Base cream)	Abundance Ratio: (Calvatia cream) / (Control)	Abundance Ratio P-Value: (Calvatia cream) / (Base cream)	Abundance Ratio P-Value: (Calvatia cream) / (Control)	Sum PEP Score
Ear1	P97426	Eosinophil cationic protein 1	22,672	21,567	1,42E-14	1,00E-17	28,07
Mmp12	P34960	Macrophage metalloelastase	17,406	9,559	1,07E-13	1,44E-09	21,06
Fcn1	O70165	Ficolin-1	13,291	2,234	3,28E-11	3,15E-02	7,10
Hmgcl	P38060	Hydroxymethylglutaryl-CoA lyase, mitochondrial	9,834	2,535	3,32E-09	1,50E-02	12,38
H2-D1	P14426	H-2 class I histocompatibility antigen, D-K alpha chain	9,29	100	4,44E-08	1,00E-17	23,46
	P01656	Ig kappa chain V-III region MOPC 70	8,825	4,236	2,32E-08	1,49E-04	35,77
Ear2	P97425	Eosinophil cationic protein 2	8,184	4,219	3,89E-09	2,20E-05	32,71
Chil4	Q91Z98	Chitinase-like protein 4	7,974	3,507	5,30E-08	9,16E-05	78,87
	P01643	Ig kappa chain V-V region MOPC 173	7,893	2,853	3,46E-07	3,38E-03	31,46
	P04940	Ig kappa chain V-VI region NQ2-17.4.1	7,309	3,376	1,31E-05	1,24E-03	14,55
Orm2	P07361	Alpha-1-acid glycoprotein 2	6,855	5,012	4,75E-07	2,42E-05	42,06
	P18528	Ig heavy chain V region 6.96	6,726	4,254	5,31E-07	1,90E-04	23,13
Ighg1	P01869	Ig gamma-1 chain C region, membrane-bound form	6,451	3,264	4,60E-05	1,72E-03	90,35
Saa2	P05367	Serum amyloid A-2 protein	6,347	3,535	4,38E-06	6,27E-04	9,87

Apcs	P12246	Serum amyloid P-component	6,341	9,682	5,44E-05	7,71E-10	51,86
Pglyrp1	O88593	Peptidoglycan recognition protein 1	6,314	3,707	5,53E-06	7,01E-05	13,07
Mpo	P11247	Myeloperoxidase	6,195	3,319	6,82E-05	1,46E-03	224,79
	P01638	Ig kappa chain V-V region L6	6,134	2,804	7,50E-05	2,59E-03	35,11
	P01657	Ig kappa chain V-III region PC 2413	6,04	4,31	7,07E-06	8,38E-05	24,36
Prxl2b	Q9DB60	Prostamide/prostaglandin F synthase	5,605	2,965	2,76E-06	8,19E-04	8,68
Prtn3	Q61096	Myeloblastin	5,5	4,083	2,06E-04	1,72E-04	22,72
Bmp1	P98063	Bone morphogenetic protein 1	5,345	2,825	2,66E-04	2,90E-03	5,56
Ambp	Q07456	Protein AMBP	5,309	3,958	2,82E-04	4,64E-05	35,36
	P01671	Ig kappa chain V-III region PC 7175	5,297	2,056	3,32E-05	2,58E-02	29,06
Ltf	P08071	Lactotransferrin	5,241	2,617	3,16E-04	1,16E-02	543,68
Epx	P49290	Eosinophil peroxidase	5,238	7,109	4,58E-06	3,12E-09	87,08
Saa1	P05366	Serum amyloid A-1 protein	5,201	3,716	1,08E-05	2,09E-04	8,87
Igkv6-17	P01633	Immunoglobulin kappa chain variable 6-17	5,168	3,413	3,57E-04	1,12E-03	8,56
Fpr2	O88536	Formyl peptide receptor 2	5,072	2,34	3,46E-05	1,60E-02	8,27
Cd177	Q8R2S8	CD177 antigen	5,025	3,012	4,55E-04	3,58E-03	137,80
Lcn2	P11672	Neutrophil gelatinase-associated lipocalin	5,024	2,874	4,56E-04	5,39E-03	57,00
Ngp	O08692	Neutrophilic granule protein	4,992	2,368	4,81E-04	2,47E-02	96,67
Hp	Q61646	Haptoglobin	4,963	4,861	5,06E-04	2,23E-05	159,04

Lcat	P16301	Phosphatidylcholine-sterol acyltransferase	4,933	3,574	2,92E-05	9,16E-04	17,75
Alox15	P39654	Polyunsaturated fatty acid lipoxygenase ALOX15	4,914	5,036	5,54E-05	3,33E-06	10,26
Retnlg	Q8K426	Resistin-like gamma	4,836	2,744	4,95E-05	8,39E-03	11,16
F5	O88783	Coagulation factor V	4,835	3,192	3,72E-05	1,99E-03	20,03
Chil3	O35744	Chitinase-like protein 3	4,831	2,782	6,35E-04	7,09E-03	161,23
Vwf	Q8CIZ8	von Willebrand factor	4,775	5,249	1,24E-04	3,07E-06	8,30
Ncf4	P97369	Neutrophil cytosol factor 4	4,772	3,744	3,82E-05	5,67E-04	33,57
Dock2	Q8C3J5	Dedicator of cytokinesis protein 2	4,766	1,556	8,22E-05	1,87E-01	7,54
Cd5l	Q9QWK4	CD5 antigen-like	4,74	3,036	7,44E-04	3,34E-03	58,28
Was	P70315	Actin nucleation-promoting factor WAS	4,732	2,086	1,16E-04	2,43E-02	4,15
C6	E9Q6D8	Complement component C6	4,677	2,528	1,97E-05	2,49E-03	64,96
Pygl	Q9ET01	Glycogen phosphorylase, liver form	4,665	2,786	8,48E-04	7,01E-03	161,84
Lyz2	P08905	Lysozyme C-2	4,603	2,692	9,45E-04	9,29E-03	23,18
Igkv9-120	P01639	Immunoglobulin kappa chain variable 9-120	4,585	1,741	4,77E-05	8,24E-02	42,60
Hspa4l	P48722	Heat shock 70 kDa protein 4L	4,561	2,108	1,02E-03	5,45E-02	49,38
Padi4	Q9Z183	Protein-arginine deiminase type-4	4,506	2,43	1,44E-04	6,10E-03	46,56
Cd14	P10810	Monocyte differentiation antigen CD14	4,5	2,704	3,14E-05	1,95E-03	50,86

Flt4	P35917	Vascular endothelial growth factor receptor 3	4,476	3,55	2,10E-04	1,79E-04	5,77
Cfh	P06909	Complement factor H	4,426	2,674	1,30E-03	9,79E-03	427,19
C3	P01027	Complement C3	4,391	2,909	1,38E-03	4,86E-03	771,35
C9	P06683	Complement component C9	4,31	2,855	1,60E-03	5,70E-03	82,29
H2ac25	Q8BFU2	Histone H2A type 3	4,288	2,197	3,07E-04	2,32E-02	16,76
Dnajc8	Q6NZB0	DnaJ homolog subfamily C member 8	4,274	3,803	8,33E-05	4,32E-05	5,25
Cyfip1	Q7TMB8	Cytoplasmic FMR1-interacting protein 1	4,193	2,587	1,98E-03	1,27E-02	85,99
Slc2a3	P32037	Solute carrier family 2, facilitated glucose transporter member 3	4,183	2,505	1,77E-04	1,52E-02	18,45
	P01644	Ig kappa chain V-V region HP R16.7	4,162	1,811	2,09E-03	8,73E-02	49,19
Nckap1l	Q8K1X4	Nck-associated protein 1-like	4,161	4,128	3,98E-04	4,50E-05	9,57
Rp2	Q9EPK2	Protein XRP2	4,147	100	3,30E-04	1,00E-17	7,46
	P18525	Ig heavy chain V region 5-84	4,128	1,455	1,94E-04	2,20E-01	15,68
Itih2	Q61703	Inter-alpha-trypsin inhibitor heavy chain H2	4,101	2,484	2,34E-03	1,74E-02	103,74
Il1rn	P25085	Interleukin-1 receptor antagonist protein	4,077	7,555	3,09E-04	7,28E-08	15,18
Itih4	A6X935	Inter alpha-trypsin inhibitor, heavy chain 4	4,064	3,245	2,50E-03	1,81E-03	205,49
Pon1	P52430	Serum paraoxonase/arylesterase 1	4,037	1,859	1,06E-04	5,21E-02	36,90

Coq6	Q8R1S0	Ubiquinone biosynthesis monooxygenase COQ6, mitochondrial	4,031	2,562	7,56E-05	4,80E-03	10,60
Sart3	Q9JLI8	Spliceosome associated factor 3, U4/U6 recycling protein	3,972	2,414	2,94E-04	9,05E-03	6,74
	P01864	Ig gamma-2A chain C region secreted form	3,926	2,453	3,24E-03	1,91E-02	61,80
Itgam	P05555	Integrin alpha-M	3,921	2,431	3,27E-03	2,04E-02	215,62
Itgb2	P11835	Integrin beta-2	3,909	2,423	3,34E-03	2,09E-02	207,63
H2-K1	P01902	H-2 class I histocompatibility antigen, K-D alpha chain	3,903	3,524	8,06E-04	3,02E-04	14,46
Serpina7	P61939	Thyroxine-binding globulin	3,863	1,995	6,47E-04	3,41E-02	7,12
Ighv1-61	P01749	Ig heavy chain V region 3	3,859	2,05	6,30E-04	4,59E-02	24,52
Ighg2b	P01867	Immunoglobulin heavy constant gamma 2B	3,858	1,979	3,68E-03	8,05E-02	85,14
Camp	P51437	Cathelicidin antimicrobial peptide	3,842	2,44	3,79E-03	1,98E-02	60,24
Ighm	P01872	Immunoglobulin heavy constant mu	3,833	2,266	3,86E-03	3,37E-02	181,45
Hpx	Q91X72	Hemopexin	3,798	2,809	4,12E-03	6,53E-03	295,94
Ahcyl2	Q68FL4	Putative adenosylhomocysteinase 3	3,798	2,235	4,12E-03	3,70E-02	22,10
Ctsg	P28293	Cathepsin G	3,775	2,707	4,30E-03	8,87E-03	46,10
Fgg	Q8VCM7	Fibrinogen gamma chain	3,763	3,308	4,40E-03	1,51E-03	278,74
	P06330	Ig heavy chain V region AC38 205.12	3,75	2,324	4,52E-03	2,83E-02	66,28

C1qc	Q02105	Complement C1q subcomponent subunit C	3,726	2,259	6,33E-04	1,21E-02	22,47
Alox5	P48999	Polyunsaturated fatty acid 5-lipoxygenase	3,718	2,719	5,81E-04	6,77E-03	16,79
Fgb	Q8K0E8	Fibrinogen beta chain	3,706	3,687	4,91E-03	5,14E-04	418,06
Pzp	Q61838	Pregnancy zone protein	3,703	2,535	4,94E-03	1,49E-02	662,84
Prg2	Q61878	Bone marrow proteoglycan	3,698	3,909	2,64E-04	1,72E-05	13,92
Mmp8	O70138	Neutrophil collagenase	3,684	2,46	5,12E-03	8,29E-03	52,03
Ppp5c	Q60676	Serine/threonine-protein phosphatase 5	3,66	1,878	2,24E-04	5,32E-02	13,20
Azgp1	Q64726	Zinc-alpha-2-glycoprotein	3,644	1,937	5,53E-03	5,52E-02	58,96
Apoh	Q01339	Beta-2-glycoprotein 1	3,644	2,037	5,53E-03	6,75E-02	132,28
C4b	P01029	Complement C4-B	3,622	2,561	5,76E-03	1,38E-02	317,86
Plg	P20918	Plasminogen	3,543	2,163	6,72E-03	4,60E-02	333,50
Itgb2l	Q3UV74	Integrin beta-2-like protein	3,523	2,312	3,84E-04	1,02E-02	46,74
C4bpa	P08607	C4b-binding protein	3,512	2,294	7,14E-03	3,10E-02	81,21
Masp2	Q91WP0	Mannan-binding lectin serine protease 2	3,5	1,572	1,14E-03	2,27E-01	20,48
B4galt1	P15535	Beta-1,4-galactosyltransferase 1	3,495	2,531	2,59E-03	1,37E-02	5,81
Gca	Q8VC88	Grancalcin	3,479	2,254	1,67E-03	2,79E-02	5,70
Serpina3m	Q03734	Serine protease inhibitor A3M	3,456	2,101	5,75E-04	1,87E-02	160,36
H2bc3	Q64475	Histone H2B type 1-B	3,431	1,985	8,36E-03	7,89E-02	70,71
Fga	E9PV24	Fibrinogen alpha chain	3,415	3,045	8,62E-03	3,25E-03	182,54

Cpn1	Q9JJN5	Carboxypeptidase N catalytic chain	3,413	2,473	6,96E-04	3,77E-03	40,64
Mmp9	P41245	Matrix metalloproteinase-9	3,388	2,333	9,10E-03	2,75E-02	69,72
Ube2v2	Q9D2M8	Ubiquitin-conjugating enzyme E2 variant 2	3,385	2,135	9,14E-03	5,01E-02	16,22
Tars1	Q9D0R2	Threonine--tRNA ligase 1, cytoplasmic	3,368	2,835	9,45E-03	6,06E-03	46,86
Ptgs1	P22437	Prostaglandin G/H synthase 1	3,362	2,719	3,87E-03	8,43E-03	10,00
Jchain	P01592	Immunoglobulin J chain	3,362	2,388	7,91E-04	6,45E-03	14,04
Fn1	P11276	Fibronectin	3,34	2,012	9,99E-03	7,28E-02	665,29
Igkv5-48	P01642	Immunoglobulin kappa variable 5-48	3,332	1,332	9,06E-04	3,31E-01	17,53
Snta1	Q61234	Alpha-1-syntrophin	3,32	3,274	1,04E-02	1,66E-03	7,93
Tap1	P21958	Antigen peptide transporter 1	3,296	3,138	2,25E-03	2,22E-03	4,92
Saa4	P31532	Serum amyloid A-4 protein	3,288	2,366	2,02E-03	6,43E-03	29,84
Orm1	Q60590	Alpha-1-acid glycoprotein 1	3,282	2,884	1,12E-02	5,23E-03	80,45
Apom	Q9Z1R3	Apolipoprotein M	3,257	2,246	1,26E-03	1,18E-02	8,01
F10	O88947	Coagulation factor X	3,21	1,921	1,16E-03	4,08E-02	26,60
H2-Q8	P14430	H-2 class I histocompatibility antigen, Q8 alpha chain	3,18	3,105	3,34E-03	8,72E-04	24,52
Eif3k	Q9DBZ5	Eukaryotic translation initiation factor 3 subunit K	3,151	1,763	2,89E-03	1,12E-01	16,13
Ncf1	Q09014	Neutrophil cytosol factor 1	3,146	2,09	1,63E-03	1,90E-02	14,09
Apod	P51910	Apolipoprotein D	3,115	1,858	3,91E-03	8,15E-02	11,48
	P01843	Ig lambda-1 chain C region	3,109	2,077	1,93E-03	2,24E-02	19,57

Afg3l2	Q8JZQ2	Mitochondrial inner membrane m-AAA protease component AFG3L2	3,106	2,221	1,64E-03	1,25E-02	16,75
Rheb	Q921J2	GTP-binding protein Rheb	3,063	2,239	1,93E-03	1,23E-02	4,61
Tcn2	O88968	Transcobalamin-2	3,053	2,272	1,76E-03	1,02E-02	39,56
Flot2	Q60634	Flotillin-2	3,027	2,791	1,87E-02	6,89E-03	16,00
B4galnt1	Q09200	Beta-1,4 N-acetylgalactosaminyltransferase 1	3,007	0,967	4,66E-03	9,85E-01	19,06
Crp	P14847	C-reactive protein	2,988	1,848	3,66E-03	6,39E-02	20,07
	P01723	Ig lambda-1 chain V region	2,982	1,729	1,97E-03	8,05E-02	18,48
Igkv12-41	P01635	Immunoglobulin kappa chain variable 12-41	2,973	2,732	2,08E-02	8,23E-03	21,76
Prss22	Q9ER10	Brain-specific serine protease 4	2,968	5,176	7,88E-03	2,97E-06	5,18
Cpb2	Q9JHH6	Carboxypeptidase B2	2,926	2,203	5,64E-03	1,23E-02	24,01
Pla2g7	Q60963	Platelet-activating factor acetylhydrolase	2,915	2,208	4,03E-03	1,21E-02	33,37
	P01807	Ig heavy chain V region X44	2,915	1,418	3,35E-03	2,47E-01	12,01
Atxn10	P28658	Ataxin-10	2,898	2,066	3,53E-03	2,64E-02	17,38
Hspb2	Q99PR8	Heat shock protein beta-2	2,898	3,044	7,37E-03	1,09E-03	5,37
Lbp	Q61805	Lipopolysaccharide-binding protein	2,855	3,021	6,40E-03	1,54E-03	15,20
Lifr	P42703	Leukemia inhibitory factor receptor	2,846	2,167	4,24E-03	1,51E-02	28,99
Sprr2d	O70555	Small proline-rich protein 2D	2,822	7,21	8,08E-03	3,48E-08	6,47

Hsd11b1	P50172	11-beta-hydroxysteroid dehydrogenase 1	2,813	2,573	3,49E-03	3,85E-03	24,92
Aldh3b1	Q80VQ0	Aldehyde dehydrogenase family 3 member B1	2,78	1,728	5,03E-03	7,40E-02	50,28
	P01797	Ig heavy chain V-III region U61	2,78	2,053	4,34E-03	2,32E-02	22,09
	P01679	Ig kappa chain V-VI region J539	2,775	0,885	8,55E-03	9,53E-01	10,61
Apoc1	P34928	Apolipoprotein C-I	2,755	2,377	3,26E-02	9,31E-03	13,20
Myoz1	Q9JK37	Myozenin-1	2,691	1,291	4,81E-03	3,72E-01	32,63
Ptpn11	P35235	Tyrosine-protein phosphatase non-receptor type 11	2,647	1,665	6,30E-03	9,91E-02	10,38
Rer1	Q9CQU3	Protein RER1	2,618	3,139	4,32E-02	2,47E-03	20,31
	P01750	Ig heavy chain V region 102	2,53	2,821	1,32E-02	7,21E-03	26,41
Aadac	Q99PG0	Arylacetamide deacetylase	2,445	3,112	2,57E-02	6,71E-04	4,49
Mpeg1	A1L314	Macrophage-expressed gene 1 protein	2,091	2,642	7,52E-02	8,48E-03	8,55
Chi3l1	Q61362	Chitinase-3-like protein 1	1,936	5,035	1,82E-01	1,44E-05	78,64
Pdlim7	Q3TJD7	PDZ and LIM domain protein 7	1,911	4,452	7,05E-02	1,43E-05	13,11
Srsf3	P84104	Serine/arginine-rich splicing factor 3	1,788	2,397	1,26E-01	6,22E-03	21,37
Il36b	Q9D6Z6	Interleukin-36 beta	1,752	4,852	1,56E-01	1,35E-05	10,19
Uox	P25688	Uricase	1,748	3,165	1,20E-01	7,48E-04	28,66
	P01878	Ig alpha chain C region	1,695	2,659	1,45E-01	3,43E-03	28,48
S100a8	P27005	Protein S100-A8	1,682	2,434	1,73E-01	5,40E-03	42,69
Trex2	Q9R1A9	Three prime repair exonuclease 2	1,55	2,676	2,76E-01	2,12E-03	38,31

Mcm4	P49717	DNA replication licensing factor MCM4	1,525	2,754	3,12E-01	4,88E-03	4,25
Tgm3	Q08189	Protein-glutamine gamma-glutamyltransferase E	1,463	3,229	4,86E-01	1,90E-03	85,99
Alox8	O35936	Polyunsaturated fatty acid lipoxygenase ALOX8	1,42	7,315	3,51E-01	2,33E-07	15,18
Ptgr2	Q8VDQ1	Prostaglandin reductase 2	1,13	0,287	7,21E-01	2,40E-03	8,39
Chkb	O55229	Choline/ethanolamine kinase	1,1	7,095	7,75E-01	1,98E-07	8,60
Rae1	Q8C570	mRNA export factor	0,891	0,224	8,05E-01	2,37E-04	6,24
Serpinb2	P12388	Plasminogen activator inhibitor 2, macrophage	0,865	0,175	6,18E-01	6,31E-07	84,03
Bcap31	Q61335	B-cell receptor-associated protein 31	0,829	0,354	6,51E-01	3,88E-03	18,04
Mtch1	Q791T5	Mitochondrial carrier homolog 1	0,825	0,291	6,39E-01	1,60E-03	8,08
Dna2	Q6ZQJ5	DNA replication ATP-dependent helicase/nuclease DNA2	0,81	4,071	6,58E-01	5,30E-05	4,25
Ptgis	O35074	Prostacyclin synthase	0,775	0,228	5,57E-01	2,29E-04	4,10
Dpysl3	Q62188	Dihydropyrimidinase-related protein 3	0,637	0,318	2,31E-01	8,39E-04	143,67
P4ha2	Q60716	Prolyl 4-hydroxylase subunit alpha-2	0,547	0,418	9,11E-02	7,35E-03	69,15
Ptma	P26350	Prothymosin alpha	0,527	0,262	1,20E-01	1,24E-03	12,05
Slc25a31	Q3V132	ADP/ATP translocase 4	0,518	0,334	1,25E-01	7,79E-03	14,53
Actn2	Q9JI91	Alpha-actinin-2	0,476	0,409	6,33E-02	8,75E-03	455,36

Lrpap1	P55302	Alpha-2-macroglobulin receptor-associated protein	0,475	0,121	7,48E-02	6,26E-08	10,36
Acsl5	Q8JZR0	Long-chain-fatty-acid--CoA ligase 5	0,464	0,37	4,15E-02	5,30E-03	63,02
Serpinh1	P19324	Serpin H1	0,463	0,382	5,47E-02	4,95E-03	219,50
P3h4	Q8K2B0	Endoplasmic reticulum protein SC65	0,461	0,352	4,59E-02	8,90E-03	20,61
Sfxn3	Q91V61	Sideroflexin-3	0,452	0,321	3,61E-02	3,49E-03	31,45
Islr	Q6GU68	Immunoglobulin superfamily containing leucine-rich repeat protein	0,441	0,325	4,67E-02	9,75E-03	15,87
Podn	Q7TQ62	Podocan	0,439	0,311	4,33E-02	4,06E-03	23,07
Ndrg2	Q9QYG0	Protein NDRG2	0,433	0,337	3,12E-02	6,15E-03	60,61
Gpx8	Q9D7B7	Probable glutathione peroxidase 8	0,418	0,31	2,52E-02	3,29E-03	11,07
Tmem214	Q8BM55	Transmembrane protein 214	0,407	0,307	2,66E-02	4,08E-03	12,55
Ufl1	Q8CCJ3	E3 UFM1-protein ligase 1	0,39	0,336	2,15E-02	6,01E-03	4,96
Serpinb12	Q9D7P9	Serpin B12	0,384	0,945	6,82E-03	9,95E-01	31,24
Aspn	Q99MQ4	Asporin	0,383	0,384	1,85E-02	5,15E-03	138,64
Dpep1	P31428	Dipeptidase 1	0,382	0,295	1,41E-02	2,48E-03	30,50
Pgm5	Q8BZF8	Phosphoglucomutase-like protein 5	0,377	0,368	1,32E-02	7,26E-03	32,45
Parva	Q9EPC1	Alpha-parvin	0,376	0,305	1,84E-02	4,20E-03	9,71
Fhl1	P97447	Four and a half LIM domains protein 1	0,374	0,335	1,60E-02	1,22E-03	56,49
Eef1a2	P62631	Elongation factor 1-alpha 2	0,365	0,392	7,25E-03	2,77E-02	126,95

Fkbp7	O54998	Peptidyl-prolyl cis-trans isomerase FKBP7	0,357	0,298	1,08E-02	2,75E-03	6,52
Rps10	P63325	Small ribosomal subunit protein eS10	0,352	0,467	3,67E-03	7,31E-02	24,84
Ndufs7	Q9DC70	NADH dehydrogenase [ubiquinone] iron-sulfur protein 7, mitochondrial	0,35	0,4	8,03E-03	2,81E-02	20,86
Ccdc47	Q9D024	PAT complex subunit CCDC47	0,348	0,329	9,60E-03	7,63E-03	9,83
Anxa5	P48036	Annexin A5	0,347	0,323	9,86E-03	1,05E-03	165,81
Trim72	Q1XH17	Tripartite motif-containing protein 72	0,345	0,337	2,92E-03	3,96E-03	86,55
Cacna2d1	O08532	Voltage-dependent calcium channel subunit alpha-2/delta-1	0,341	0,369	6,58E-03	1,42E-02	78,72
Adh7	Q64437	All-trans-retinol dehydrogenase [NAD(+)] ADH7	0,339	0,408	8,46E-03	6,25E-03	68,81
Elovl4	Q9EQC4	Very long chain fatty acid elongase 4	0,336	0,319	8,51E-03	8,45E-03	9,61
Hhatl	Q9D1G3	Protein-cysteine N-palmitoyltransferase HHAT-like protein	0,332	0,487	5,14E-03	8,76E-02	37,43
Ncln	Q8VCM8	BOS complex subunit NCLN	0,33	0,232	6,03E-03	6,62E-04	9,25
Tpm2	P58774	Tropomyosin beta chain	0,329	0,32	6,90E-03	8,98E-04	185,79
Sdhc	Q9CZB0	Succinate dehydrogenase cytochrome b560 subunit, mitochondrial	0,324	0,499	6,47E-03	1,20E-01	8,16

Sypl2	O89104	Synaptophysin-like protein 2	0,317	0,502	5,60E-03	1,12E-01	14,40
Mcpt4	P21812	Mast cell protease 4	0,315	0,415	1,28E-03	2,71E-02	42,26
Echdc1	Q9D9V3	Ethylmalonyl-CoA decarboxylase	0,312	0,279	1,00E-03	2,49E-04	80,97
Myh1	Q5SX40	Myosin-1	0,306	0,174	4,13E-03	5,95E-07	1257,92
Sec63	Q8VHE0	Translocation protein SEC63 homolog	0,302	0,278	4,41E-03	1,50E-03	9,68
Psapl1	Q8C1C1	Proactivator polypeptide-like 1	0,299	0,48	3,96E-03	9,27E-02	13,85
Cmbl	Q8R1G2	Carboxymethylenebutenolidase homolog	0,295	0,267	4,17E-03	2,04E-03	7,38
Shtn1	Q8K2Q9	Shootin-1	0,293	0,302	3,06E-03	3,52E-03	7,91
Myom3	A2ABU4	Myomesin-3	0,293	0,232	2,48E-03	3,63E-04	74,66
Alox12e	P55249	Polyunsaturated fatty acid (12S)/(13S)-lipoxygenase, epidermal-type	0,287	0,32	1,22E-03	2,15E-03	26,99
Macrod1	Q922B1	ADP-ribose glycohydrolase MACROD1	0,285	0,305	2,15E-03	3,71E-03	25,50
S100a16	Q9D708	Protein S100-A16	0,282	0,752	9,53E-04	5,45E-01	16,55
Lclat1	Q3UN02	Lysocardiolipin acyltransferase 1	0,277	0,371	1,86E-03	2,46E-02	5,67
Maob	Q8BW75	Amine oxidase [flavin-containing] B	0,275	0,403	1,80E-03	3,44E-02	14,56
Hdhd2	Q3UGR5	Haloacid dehalogenase-like hydrolase domain-containing protein 2	0,272	0,228	1,83E-03	2,21E-04	7,73

Stim1	P70302	Stromal interaction molecule 1	0,271	0,276	1,31E-03	1,63E-03	13,96
Rps27l	Q6ZWY3	Small ribosomal subunit protein eS27-like	0,27	0,426	1,22E-03	4,40E-02	13,67
Serpina3b	Q8BYY9	Serine protease inhibitor A3B	0,269	0,067	1,42E-03	2,40E-12	8,90
Dnajc7	Q9QYI3	DnaJ homolog subfamily C member 7	0,266	0,171	9,63E-04	2,95E-05	8,34
Tnnt3	Q9QZ47	Troponin T, fast skeletal muscle	0,264	0,295	1,36E-03	1,76E-04	96,78
Rbm39	Q8VH51	RNA-binding protein 39	0,26	0,213	3,00E-04	2,39E-05	9,26
Gstk1	Q9DCM2	Glutathione S-transferase kappa 1	0,256	0,516	9,90E-04	1,91E-01	6,90
Kdsr	Q6GV12	3-ketodihydrosphingosine reductase	0,256	0,269	1,22E-03	1,20E-03	6,41
Lhpp	Q9D7I5	Phospholysine phosphohistidine inorganic pyrophosphate phosphatase	0,254	0,171	1,16E-03	8,13E-06	5,84
Skic3	F8VPK0	Superkiller complex protein 3	0,248	0,227	5,43E-04	1,83E-04	10,18
Dbt	P53395	Lipoamide acyltransferase component of branched-chain alpha-keto acid dehydrogenase complex, mitochondrial	0,246	0,238	8,22E-04	3,03E-04	8,00
Apobec2	Q9WV35	C->U-editing enzyme APOBEC-2	0,245	0,265	6,36E-04	1,92E-03	20,35
Mybph	P70402	Myosin-binding protein H	0,24	0,311	2,47E-04	4,55E-03	82,65
Mb	P04247	Myoglobin	0,239	0,18	6,43E-05	1,65E-07	65,61
Acta1	P68134	Actin, alpha skeletal muscle	0,239	0,367	1,32E-04	5,55E-03	400,78

Padi2	Q08642	Protein-arginine deiminase type-2	0,236	0,875	4,77E-04	9,54E-01	45,41
Tnni2	P13412	Troponin I, fast skeletal muscle	0,235	0,253	5,79E-05	1,60E-04	48,42
Fnta	Q61239	Protein farnesyltransferase/geranylgeranyltransferase type-1 subunit alpha	0,225	0,213	4,78E-04	1,27E-04	9,13
Parp1	P11103	Poly [ADP-ribose] polymerase 1	0,211	0,159	1,70E-04	4,34E-06	12,63
Anxa4	P97429	Annexin A4	0,208	0,448	6,35E-05	3,83E-02	75,30
Cma1	P21844	Chymase	0,199	0,241	2,03E-05	4,44E-04	36,64
Cyp2f2	P33267	Cytochrome P450 2F2	0,196	0,19	2,51E-05	4,49E-05	46,50
Myh11	O08638	Myosin-11	0,193	0,19	9,55E-05	3,38E-05	77,37
Cacnb1	Q8R3Z5	Voltage-dependent L-type calcium channel subunit beta-1	0,173	0,362	1,39E-05	1,49E-02	15,07
Mgll	O35678	Monoglyceride lipase	0,172	0,172	7,24E-07	1,57E-06	46,65
Acsbg1	Q99PU5	Long-chain-fatty-acid--CoA ligase ACSBG1	0,169	0,161	1,46E-05	8,90E-06	10,28
Far2	Q7TNT2	Fatty acyl-CoA reductase 2	0,161	0,112	1,19E-06	4,21E-10	42,17
Lamtor3	O88653	Ragulator complex protein LAMTOR3	0,154	0,289	3,27E-06	2,32E-03	14,24
Cstf1	Q99LC2	Cleavage stimulation factor subunit 1	0,152	0,398	7,41E-06	2,83E-02	8,01
Myot	Q9JIF9	Myotilin	0,151	0,176	5,73E-06	1,28E-05	44,62

Sdr16c6	Q05A13	Short-chain dehydrogenase/reductase family 16C member 6	0,15	0,129	1,63E-06	8,25E-08	41,62
Akr1e2	Q9DCT1	1,5-anhydro-D-fructose reductase	0,147	0,184	3,03E-06	3,88E-05	6,13
Lamb2	Q61292	Laminin subunit beta-2	0,14	0,128	1,22E-06	2,96E-07	13,38
Phkg1	P07934	Phosphorylase b kinase gamma catalytic chain, skeletal muscle/heart isoform	0,134	0,123	6,61E-08	2,62E-07	13,48
Dhcr24	Q8VCH6	Delta(24)-sterol reductase	0,13	0,254	7,04E-07	7,18E-04	5,38
Hal	P35492	Histidine ammonia-lyase	0,122	0,154	6,96E-07	3,76E-08	114,73
Sec14l4	Q8R0F9	SEC14-like protein 4	0,122	0,115	1,85E-07	1,51E-07	25,34
Cox7a1	P56392	Cytochrome c oxidase subunit 7A1, mitochondrial	0,087	0,104	1,78E-09	8,06E-09	10,43
Flg2	Q2VIS4	Filaggrin-2	0,035	0,04	1,00E-17	2,22E-16	24,24
Mccc2	Q3ULD5	Methylcrotonoyl-CoA carboxylase beta chain, mitochondrial	0,01	0,01	1,00E-17	1,00E-17	19,06
Tmod1	P49813	Tropomodulin-1	0,01	0,01	1,00E-17	1,00E-17	5,78
Sgcb	P82349	Beta-sarcoglycan	0,01	0,01	1,00E-17	1,00E-17	9,05
Reep6	Q9JM62	Receptor expression-enhancing protein 6	0,01	0,01	1,00E-17	1,00E-17	6,95
Camk2a	P11798	Calcium/calmodulin-dependent protein kinase type II subunit alpha	0,01	0,01	1,00E-17	1,00E-17	17,91

Tmem38a	Q3TMP8	Trimeric intracellular cation channel type A	0,01	0,01	1,00E-17	1,00E-17	4,63
Gnai1	B2RSH2	Guanine nucleotide-binding protein G(i) subunit alpha-1	0,01		1,00E-17		39,13
Dhrs7c	Q8CHS7	Dehydrogenase/reductase SDR family member 7C	0,01	0,01	1,00E-17	1,00E-17	9,98
Hrnr	Q8VHD8	Hornerin	0,01	0,01	1,00E-17	1,00E-17	9,42
Hmgcs2	P54869	Hydroxymethylglutaryl-CoA synthase, mitochondrial	0,01	0,01	1,00E-17	1,00E-17	10,91
