# Supplementary figures and images for: Integrative In Vivo and Proteomic Analysis of a Bovistella utriformis Polysaccharide Formulation Reveals Mechanisms of Enhanced Skin Wound Healing
Source: Molecules. 2026 Apr 8;31(8):1233. doi: 10.3390/molecules31081233 (PMC13119201; doi:10.3390/molecules31081233)

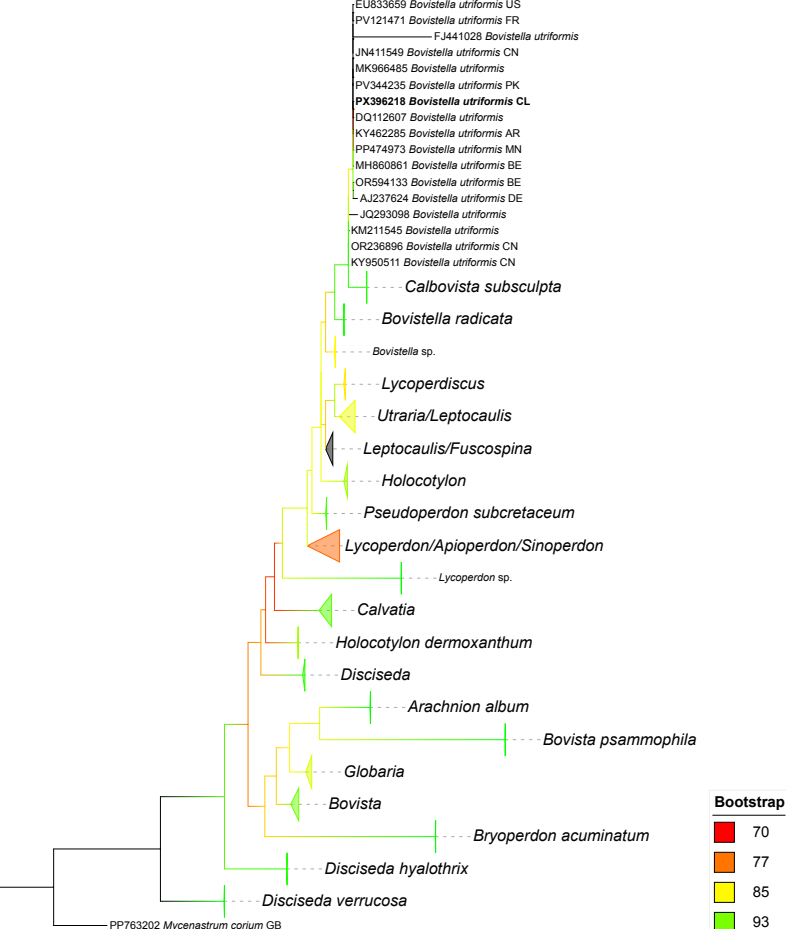

Tree scale: 0.1

Supplement: Supplementary file 1 [file molecules-31-01233-s001.zip › Supplementary Material S1 of phylogenitic/collapsedTree1.pdf]
