# Supplementary material for: Integrative In Vivo and Proteomic Analysis of a Bovistella utriformis Polysaccharide Formulation Reveals Mechanisms of Enhanced Skin Wound Healing
Source: Molecules. 2026 Apr 8;31(8):1233. doi: 10.3390/molecules31081233 (PMC13119201; doi:10.3390/molecules31081233)
Supplement: Supplementary file 1 [file composition.pdf › supplementary material S2 de la crema base/composition.pdf]

Camassia ECO s.l.
B04785176
Calle V, Nave 26, Pol.Ind.La Redonda
04710 Santa Maria del Águila
Almeria, España
Telf. +34 950 57 61 62 info@cremas-caseras.es

Ficha Técnica

Producto: Crema Base Certificada Ecológica Fecha: 08/06/2021

INCI: Aloe barbadensis Leaf Juice*, Caprylic/capric Triglyceride, Glycerin**, Cetyl Alcohol, Cetearyl Olivate, Butyrospermum Parkii Butter*, Sorbitan Olivate, Glyceryl Caprylate, Tocopherol, Xanthan Gum, Glyceryl Undecylenate, Phytic Acid, Aqua, Benzyl Alcohol.

INCI	%
Aloe Barbadensis Leaf Juice	75-77%
CAPRYLIC TRIGLYCERIDE	7,5-9,0 %
Glycerin	3,0-3,5 %
Cetyl Alcohol	3,0-3,5 %
Cetearyl olivate	2,5-3,0%
Butyrospermum Parkii Butter	2,0-2,5 %
Sorbitan Olivate	1,5-2,0 %
Glyceryl Caprylate	0,4-0,5 %
Tocopherol	0,2-0,3%
Xanthan gum	0,8-1,2 %
Glyceryl undecilate	0,1-0,2%
Phytic Acid	0,05-0,1%
Aqua	c.s.p
Benzyl Alcohol	0,4-0,5 %

CREMA BASE

- TABLA DE ESPECIFICACIONES TÉCNICAS

CONFIDENCIAL

PARÁMETROS	ESPECIFICACIÓN
Aspecto	Emulsión
Color	Blanco
Olor	Característico
Densidad	0,956-1,056 g/ml
PH	5,3-5,6
Microbiología	• Mesófilos Aerobios: <500 UFC

	• Hongos y levaduras: < 500 UFC<br>• Ausencia de microorganismos patógenos; E. Coli, P. Aeruginosa, S. Aureus, Candida Albicans.

Exención de responsabilidad: Este documento, o cualquier respuesta o información proporcionada aquí por Camassia ECO sl,no constituye una obligación legalmente vinculante de Camassia ECO sl. Mientras que las descripciones, los diseños, los datos y la información contenidos en este documento se presentan de buena fé y se consideran precisos; se proporcionan únicamente para su orientación porque muchos factores pueden afectar el procesamiento o la aplicación / uso. Le recomendamos que realice pruebas para determinar la idoneidad de un producto para su propósito particular antes de usar. No exime a nuestros clientes de la obligación de realizar una inspección completa de los productos en el momento de la entrega o de cualquier otra obligación.
